# Elevated prelimbic cortex-to-basolateral amygdala circuit activity mediates comorbid anxiety-like behaviors associated with chronic pain

**DOI:** 10.1172/JCI166356

**Published:** 2023-05-01

**Authors:** Feng Gao, Jie Huang, Guo-Bin Huang, Qiang-Long You, Shan Yao, Shen-Ting Zhao, Jian Liu, Cui-Hong Wu, Gui-Fu Chen, Shi-Min Liu, Zongyan Yu, Yan-Ling Zhou, Yu-Ping Ning, Shenquan Liu, Bing-Jie Hu, Xiang-Dong Sun

**Affiliations:** 1 Department of Neurology, Institute of Neuroscience, Key Laboratory of Neurogenetics and Channelopathies of Guangdong Province and the Ministry of Education of China, and Emergency Department of the Second Affiliated Hospital, School of Basic Medicine, Guangzhou Medical University, Guangzhou, China.; 2 Department of Physiology, School of Basic Medicine, Guangdong Medical University, Zhanjiang, China.; 3 Department of Physiology, School of Basic Medicine, Guangzhou Medical University, Guangzhou, China.; 4 Department of Psychiatry, Affiliated Brain Hospital of Guangzhou Medical University, Guangzhou, China.; 5 School of Mathematics, South China University of Technology, Guangzhou, China.

**Keywords:** Neuroscience, Behavior, Cytokines, Pain

## Abstract

Chronic pain can cause both hyperalgesia and anxiety symptoms. However, how the two components are encoded in the brain remains unclear. The prelimbic cortex (PrL), a critical brain region for both nociceptive and emotional modulations, serves as an ideal medium for comparing how the two components are encoded. We report that PrL neurons projecting to the basolateral amygdala (PrL^BLA^) and those projecting to the ventrolateral periaqueductal gray (PrL^l/vlPAG^) were segregated and displayed elevated and reduced neuronal activity, respectively, during pain chronicity. Consistently, optogenetic suppression of the PrL-BLA circuit reversed anxiety-like behaviors, whereas activation of the PrL-l/vlPAG circuit attenuated hyperalgesia in mice with chronic pain. Moreover, mechanistic studies indicated that elevated TNF-α/TNFR1 signaling in the PrL caused increased insertion of GluA1 receptors into PrL^BLA^ neurons and contributed to anxiety-like behaviors in mice with chronic pain. Together, these results provide insights into the circuit and molecular mechanisms in the PrL for controlling pain-related hyperalgesia and anxiety-like behaviors.

## Graphical abstract

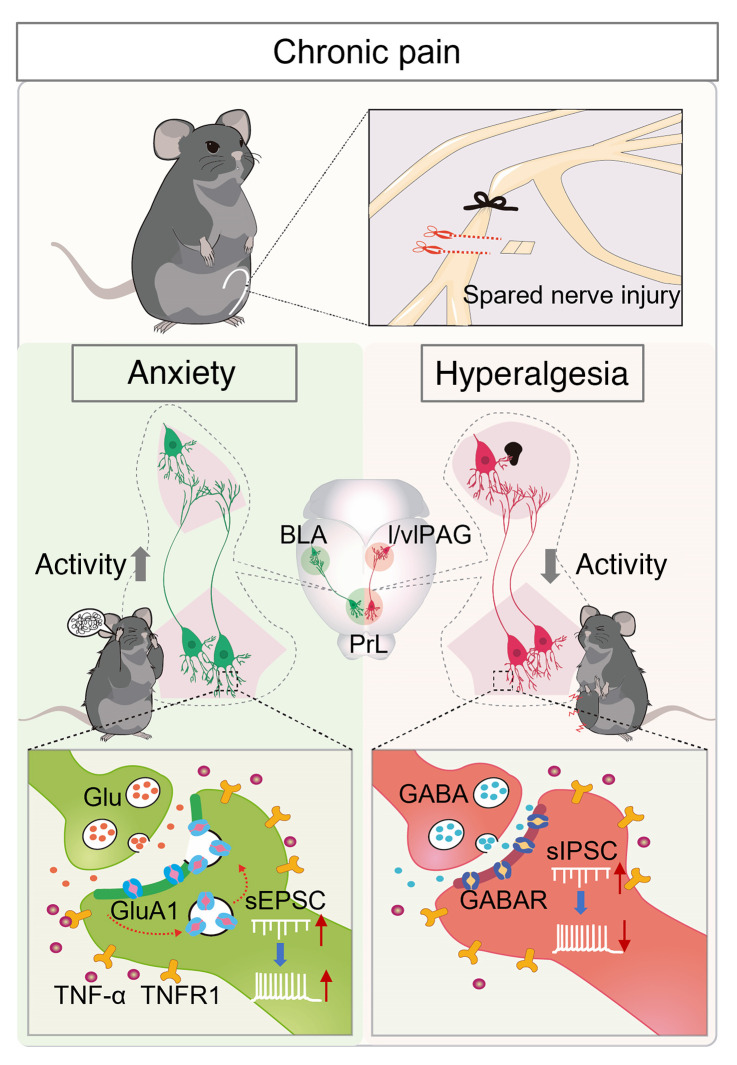

## Introduction

Chronic pain is a debilitating disorder affecting more than 30% of the world’s population (1). One of the potential reasons that chronic pain difficult to treat is that patients suffering from chronic pain frequently perceive profound negative emotional changes, such as anxiety, which, in turn, aggravate sensory pain symptoms (2). Achieving a better understanding of the neuronal circuitry and molecular mechanisms underlying pain hypersensitivity and comorbid anxiety is vital to the development of effective therapeutic strategies for relieving suffering in patients with chronic pain.

Pain is an unpleasant experience that involves sensory, motivational, and affective components (3). Nociceptive information is relayed through the peripheral sensory nervous system into high-order brain regions for pain perception, and, in parallel, interacts with affective pathways, the maladaptive activation of which is implicated in emotional dysfunctions during pain chronicity (4). Accumulating evidence has indicated that the medial prefrontal cortex (mPFC), a crucial region for top-down control of sensory and emotional processes, plays a fundamental role in the regulation of both pain hypersensitivity and emotional deficits in chronic pain (5, 6). For instance, the neuronal activity in the prelimbic cortex (PrL), a major component of the mPFC, was reported to be reduced in both neuropathic and inflammatory pain animal models (7–9). Activation of all of the PrL neurons, or a particular neuron population projecting to the ventrolateral periaqueductal gray (vlPAG), ameliorated the hyperalgesia phenotypes in mice with chronic pain (9–12). These observations indicate that weakened PrL neuronal activity contributes to heightened nociceptive processing during chronic pain. In contrast, ample evidence suggests that the PrL became hyperactive at a high-anxiety state (13). Pharmacological activation and inhibition of PrL neurons could induce anxiogenic and anxiolytic effects, respectively (14–16). It is known that mPFC projection neurons send immense outputs to the basolateral amygdala (BLA), a region critical for emotion control (17, 18). A recent study reports an enhanced activity of the mPFC-BLA circuit and consequent anxiety-like behaviors caused by chronic resistant stress (19), suggesting a pivotal role of the mPFC-BLA circuit in mediating negative affective behaviors. Considering the comorbidity between hyperalgesia and anxiety symptoms during chronic pain, these seemingly discordant observations of neuronal activity in the PrL suggest that circuit-specific dysfunctions of PrL neurons may individually influence hyperalgesia and anxiety symptoms under chronic pain. Yet, whether and how the circuit-specific changes under chronic pain occur in the PrL — and the underlying molecular mechanisms — remain to be elucidated.

Using viral tracing, in-vivo 2-photon (2P) calcium (Ca^2+^) imaging, optogenetic, electrophysiological, and behavioral approaches, we demonstrate that 2 different neuronal populations in the PrL, which project to l/vlPAG and BLA regulate hyperalgesia and anxiety-like behaviors, respectively, in a mouse model of chronic pain. Subsequent mechanistic experiments indicate that increased TNF-α signaling caused an upregulation of postsynaptic expression of GluA1 receptors to enhance the neuronal activity of PrL pyramidal neurons that innervate BLA (PrL^BLA^). Collectively, our study provides what we believe to be compelling evidence to reveal the circuit and molecular mechanisms underlying the comorbidity of hyperalgesia and anxiety in chronic pain.

## Results

### Peripheral nerve injury induces time-dependent and projection-specific neuronal activity changes in the PrL.

**Figure 1:**
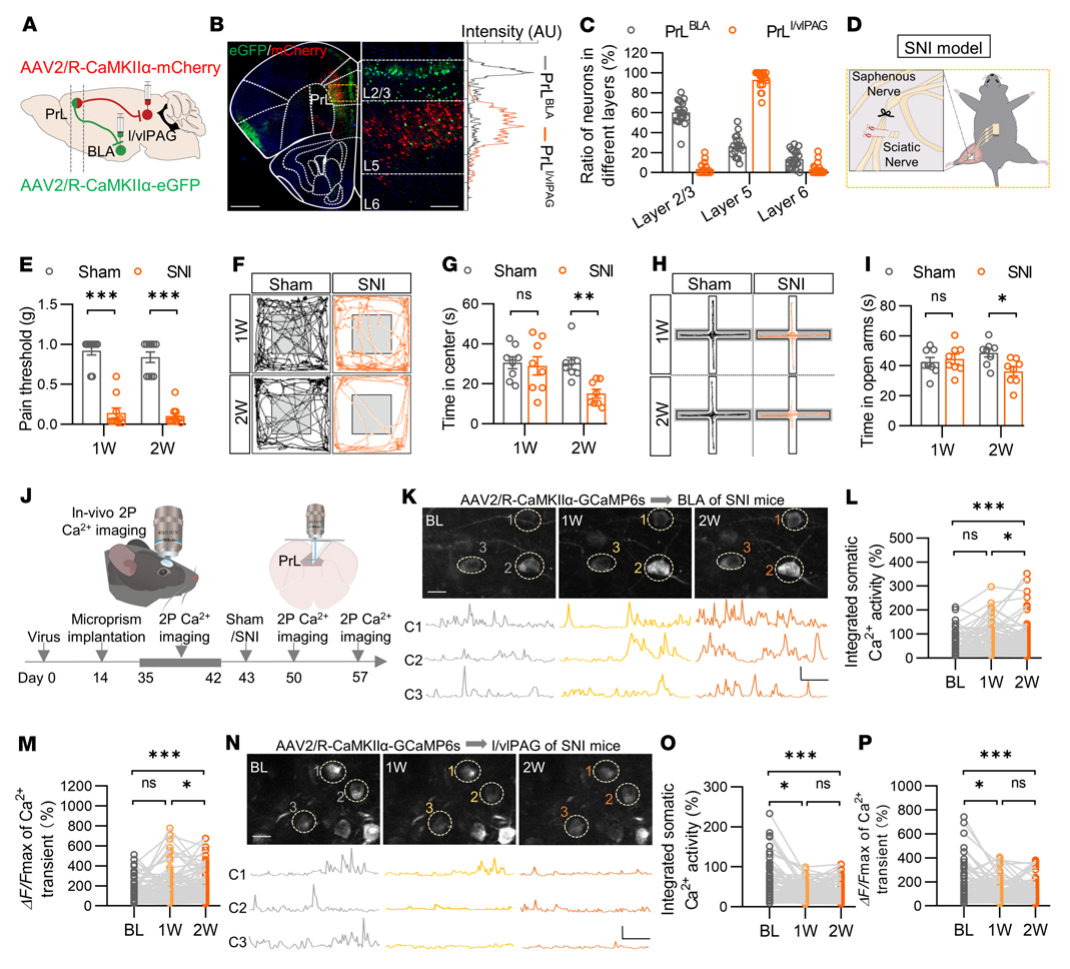
Peripheral nerve injury induces time-dependent and projection-specific changes in neuronal activity in the PrL. (**A**) Schematic for viral injections. (**B**) Expressions of eGFP and mCherry in the PrL. Left, half brain section containing the PrL region; middle, enlarged indicated portion from the left image that was rotated counterclockwise 90°; right, intensity distribution of green (PrL^BLA^) and red (PrL^l/vlPAG^) fluorescence throughout the different layers in the PrL. a.u., arbitrary unit. Scale bars: 600 μm (left) and 100 μm (middle). (**C**) Ratios of PrL^BLA^ and PrL^l/vlPAG^ neurons. *n* = 20 slices from 5 mice. (**D**) Schematic for SNI surgery. (**E**) Decreased mechanical pain threshold in SNI mice. *n* = 10 mice per group. (**F** and **H**) Representative traces of mice travel in OFT (**F**) and in EPM (**H**). (**G**) Reduced time in center in SNI mice. *n* = 8 mice per group. (**I**) Reduced time in open arms in SNI mice. *n* = 8 mice per group. (**J**) Time scheme for in-vivo 2P Ca^2+^ imaging on awake mice. (**K**) Representative somatic Ca^2+^ fluorescent images of PrL^BLA^ neurons before (BL), 1 week (1W) and 2 weeks (2W) in SNI mice. Bottom, Ca^2+^ fluorescent traces from the numbered neurons (C1, C2, C3), which are circled in the upper images. Scale bars: 10 μm (upper), 500% *ΔF/F* and 20 s (bottom). (**L** and **M**) Increased integrated somatic Ca^2+^ activity (**L**) and peak amplitude (**M**) of Ca^2+^ transients of PrL^BLA^ neurons in SNI mice. *n* = 131 neurons from 6 mice. (**N**) Representative somatic Ca^2+^ fluorescent images of PrL^l/vlPAG^ neurons. Bottom, Ca^2+^ fluorescent traces from the numbered neurons (C1, C2, C3), which are circled in the upper images. Scale bars: 10 μm (upper), 500% *ΔF/F* and 20 s (bottom). (**O** and **P**) Decreased integrated somatic Ca^2+^ activity (**O**) and peak amplitude (**P**) of Ca^2+^ transients of PrL^l/vlPAG^ neurons in SNI mice. *n* = 104 neurons from 5 mice. Data shown as mean ± SEM or aligned dot plots. *P < 0.05, **P < 0.01, ***P < 0.001. 2-way repeated measures ANOVA followed by posthoc Šidák’s test (**E**, **G**, and **I**); Friedman test with posthoc Dunn’s test (**L**, **M**, **O**, and **P**).

To identify excitatory neurons in the PrL that project to BLA (PrL^BLA^) and l/vlPAG (PrL^l/vlPAG^), we injected retrograde adeno-associated viral vectors (AAV2/Rs) carrying eGFP or mCherry controlled by the CaMKIIα promoter into the BLA and l/vlPAG, respectively (Figure 1A and Supplemental Figure 1, A–D; supplemental material available online with this article; https://doi.org/10.1172/JCI166356DS1). 3 weeks later, we compared the distribution of PrL^BLA^ and PrL^l/vlPAG^ neurons in the PrL. Most PrL^BLA^ and PrL^l/vlPAG^ neurons were costained with a CaMKIIα antibody (Supplemental Figure 1, E–G), confirming their excitatory-neuron identity. Intriguingly, these projection-specific neurons were barely colocalized (Figure 1B). Notably, approximately 93% of PrL^l/vlPAG^ excitatory neurons were restricted in layer 5 of the PrL, with 3.6% in layer 2/3. In contrast, only 26.4% of PrL^BLA^ excitatory neurons were localized in layer 5, with 60.2% in layer 2/3 (Figure 1C). These observations are consistent with previous reports (6, 10, 12, 20–23) and indicate that PrL^BLA^ and PrL^l/vlPAG^ excitatory neurons exhibit a layer-specific and nonoverlapping distribution pattern.

To delineate the impacts of chronic pain on the activity of these projection-specific neurons in the PrL, we utilized a mouse model of neuropathic pain, in which chronic pain was induced by spared nerve injury (SNI) (24, 25) (Figure 1D). The SNI mice exhibited obvious hyperalgesia symptoms as the mechanical thresholds for pain perception were drastically reduced after surgery (Figure 1E). While these mice traveled a similar distance in the open field test (OFT) (Supplemental Figure 1H), they spent a shorter time in the center area of the arena 2 weeks rather than 1 week after surgery when compared with sham control mice (Figure 1, F and G). In addition, the time in the open arms and the probability of open-arm entry in the elevated plus-maze test (EPM) were decreased when compared with sham controls 2 weeks, but not 1 week, after surgery (Figure 1, H and I and Supplemental Figure 1I). These results suggest a time-dependent development of anxiety-like behaviors during pain chronicity. We then performed in-vivo 2P Ca^2+^ imaging to longitudinally monitor the real-time activity of these neurons in head-fixed awake mice following surgery (Figure 1J). A retrograde AAV carrying genetically encoded Ca^2+^ indicator GCaMP6s was injected into the BLA or l/vlPAG region, and a right-angle microprism was implanted into the contralateral hemisphere to enable Ca^2+^ imaging of the ipsilateral PrL region (Supplemental Figure 2, A–D). Spontaneous Ca^2+^ transients of PrL^BLA^ or PrL^l/vlPAG^ neurons were recorded 3 weeks later. We found that neither the integrated somatic Ca^2+^ activity nor the peak amplitude of Ca^2+^ transients was changed in PrL^BLA^ or PrL^l/vlPAG^ neurons of sham mice at different time points after surgery (Supplemental Figure 2, E–H and Supplemental Videos 1 and 2), indicating the stability of Ca^2+^ activity over time. In contrast, both parameters were increased in PrL^BLA^ neurons of SNI mice 2 weeks after surgery, although there was no difference 1 week after surgery (Figure 1, K–M and Supplemental Video 3). Differently, the integrated somatic Ca^2+^ activity and the peak amplitude of Ca^2+^ transients were decreased in PrL^l/vlPAG^ neurons of SNI mice 1 week and 2 weeks after surgery (Figure 1, N–P and Supplemental Video 4). Altogether, these data demonstrate time-dependent and projection-specific changes of neuronal activity in the PrL in chronic pain.

### Optogenetic manipulations of the PrL-BLA circuit modulate anxiety-like behaviors but not hyperalgesia in mice with chronic pain.

**Figure 2:**
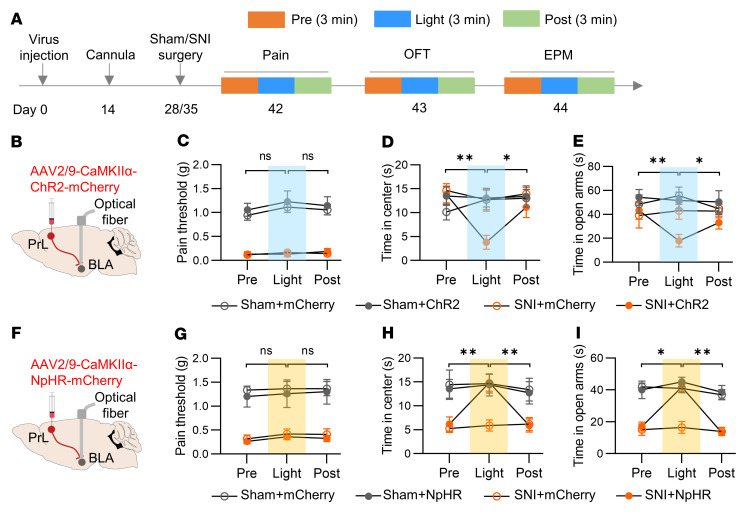
Optogenetic manipulations of the PrL-BLA circuit modulates anxiety-like behaviors but not hyperalgesia in mice with chronic pain. (**A**) Experimental time scheme. (**B**) Schematic of injection of AAV-CaMKIIα-ChR2-mcherry into the PrL and implantation of optical cannula into the BLA. (**C**) Unchanged pain threshold by activation of PrL input in BLA. *n* = 7 mice per group. (**D** and **E**) Decreased time in center in OFT (**D**) and time in open arms in EPM (**E**) by activation of PrL input in BLA of SNI mice. *n* = 7 and 9 mice for sham and SNI groups. (**F**) Schematic of injection of AAV-CaMKIIα-NpHR-mcherry into the PrL and implantation of optical cannula into the BLA. (**G**) Unchanged pain threshold by inhibition of PrL input in BLA. *n* = 6 and 7 mice for sham and SNI groups. (**H**) Increased time in center in OFT by inhibition of PrL input in BLA. *n* = 7 mice per group. (**I**) Increased time in open arms in EPM by inhibition of PrL input in BLA. *n* = 7 mice per group. Data shown as mean ± SEM. *P < 0.05, **P < 0.01. Repeated 1-way ANOVA followed by posthoc Tukey’s test (**C**–**E** and **G**–**I**).

To investigate the roles of projection-specific circuits in the chronic pain model, we attempted to manipulate the individual circuits using optogenetics and to test the pain threshold and anxiety-like behaviors (Figure 2A). We first targeted the PrL-BLA circuit utilizing optogenetics. Channelrhodopsin-2 (ChR2) was virally expressed in the PrL under the control of the CaMKIIα promoter (Supplemental Figure 3A). Current-clamp analysis on brain slices showed faithful induction of time-locked action potentials in ChR2-expressing PrL neurons with blue laser pulses (Supplemental Figure 3, B–D). Moreover, stimulation of ChR2-positive fibers in the BLA evoked short-latency excitatory postsynaptic currents (eEPSCs in the BLA neurons (Supplemental Figure 3, E–H). The eEPSCs could be blocked by tetrodotoxin (TTX) and rescued by further treatment of 4-aminopyridine (4-AP), a potassium channel blocker (Supplemental Figure 3, G and H). These observations indicate a monosynaptic connection between PrL excitatory neurons and BLA neurons, which is in line with previous reports (19, 26).

To examine whether activation of the PrL-BLA circuit could promote anxiety-like behaviors in mice with chronic pain, we injected the ChR2-expressing viral vectors into the PrL and implanted an optic cannula into the BLA (Figure 2B). The PrL-BLA circuit was activated by blue laser lights, and the pain threshold and anxiety-like behaviors were examined 1 week after SNI surgery when the mice hadn’t displayed anxiety-like phenotypes. We found that blue-light stimulation exhibited minimal effects on the pain threshold, suggesting a dispensable role of the PrL-BLA circuit in pain perception (Figure 2C). However, it decreased the time in the center area in the OFT and the time in the open arms in the EPM in SNI mice expressing ChR2 (SNI+ChR2), whereas those parameters were unaltered in sham mice expressing ChR2 (Sham+ChR2), sham mice expressing mCherry (Sham+mCherry), or SNI mice expressing mCherry (SNI+mCherry) (Figure 2, D and E). These results suggest that activation of the PrL-BLA circuit promotes anxiety-like behaviors with little effect on pain perception.

To investigate whether inhibition of the PrL-BLA circuit could reverse anxiety symptoms in mice with chronic pain, we injected halorhodopsin-expressing (NpHR-expressing) viral vectors into the PrL (Supplemental Figure 4A). Continuous yellow light illumination reliably suppressed the firing of PrL pyramidal neurons (Supplemental Figure 4, B and C). In addition, optical inhibition of PrL afferent terminals in the BLA decreased the frequency but not the amplitude of spontaneous excitatory postsynaptic currents (sEPSCs), with little effect on spontaneous inhibitory postsynaptic currents (sIPSCs) (Supplemental Figure 4, D–N). These observations indicate a successful inhibition of the PrL-BLA circuit by yellow light. Remarkably, optical inhibition of the PrL-BLA circuit exhibited little effect on the pain threshold but increased the time in the center in the OFT and the time in the open arms in the EPM in SNI mice expressing NpHR, rather than control mice expressing mCherry or sham mice with/without NpHR expression at 2 weeks after surgery (Figure 2, F–I). Together, these results demonstrate a pivotal role of the PrL-BLA circuit in regulating anxiety-like behaviors but not hyperalgesia in mice with chronic pain. This notion is strengthened by the results that a proportion of PrL^BLA^ neurons from both sham and SNI mice exhibited increased Ca^2+^ activity in response to fox urine and foot shock (Supplemental Figure 5), 2 widely used anxiogenic stimuli (27, 28). Notably, the proportions of these responsive PrL^BLA^ neurons were increased in SNI mice compared with those in sham mice (18.85% to 30.87% for fox urine; 22.64% to 46.36% for foot shock). In addition, the integrated somatic Ca^2+^ activity and the peak amplitude of Ca^2+^ transients were also elevated (Supplemental Figure 5). These observations indicate an adaptation in the subpopulation composition and activities of PrL^BLA^ neurons during pain chronicity.

### The PrL-l/vlPAG circuit is required for modulation of pain perception but not anxiety-like behaviors.

**Figure 3:**
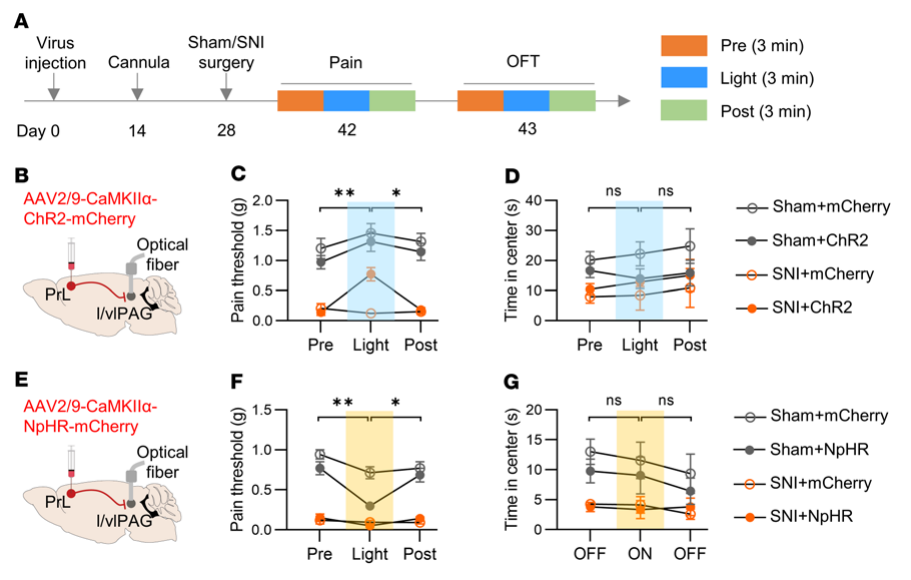
The PrL-l/vlPAG circuit is required for modulation of pain perception but not anxiety-like behaviors. (**A**) Experimental time scheme. (**B**) Schematic of injection of AAV-CaMKIIα-ChR2-mcherry into the PrL and implantation of optical cannula into the l/vlPAG. (**C**) Increased pain threshold by activation of PrL input in l/vlPAG. *n* = 7 mice per group. (**D**) Unchanged time in center in OFT by activation of PrL input in l/vlPAG. *n* = 7 mice per group. (**E**) Schematic of injection of AAV-CaMKIIα-NpHR-mcherry into the PrL and implantation of optical cannula into the l/vlPAG. (**F**) Decreased pain threshold by inhibition of PrL input in l/vlPAG. *n* = 7 mice per group. (**G**) Unchanged time in center in OFT by inhibition of PrL input in l/vlPAG. *n* = 7 mice per group. Data shown as mean ± SEM. *P < 0.05, **P < 0.01. Friedman test followed by posthoc Dunn’s test (**C**, **D**, and **F**); Repeated 1-way ANOVA followed by posthoc Tukey’s test (**G**).

Previous studies have reported a role of the mPFC-PAG circuit in regulating pain hypersensitivity in chronic pain (10, 12, 29). However, results regarding whether this circuit regulates anxiety-like behaviors remain complicated. We then attempted to dissect the involvement of the PrL-l/vlPAG circuit in hyperalgesia and comorbid anxiety (Figure 3A). Extensive fibers were observed in the l/vlPAG region of mice injected with AAV expressing mCherry in the PrL (Supplemental Figure 6A). A single blue laser pulse elicited an EPSC, which was blocked and rescued by TTX and 4-AP treatments, respectively (Supplemental Figure 6, B–D), indicating a monosynaptic input from PrL neurons to l/vlPAG neurons. Behaviorally, optical stimulation of PrL input terminals in the l/vlPAG dramatically increased the pain threshold in both the sham and SNI mice expressing ChR2, but not in those expressing mCherry (Figure 3, B and C). These observations demonstrate a suppressive effect of the PrL-l/vlPAG circuit on pain perception, in line with previous reports (10, 12). However, blue laser pulses failed to affect the time in the center in the OFT in mice from either group (Figure 3D). These data suggest that activation of the PrL-l/vlPAG circuit does not modulate anxiety-like behaviors. In turn, optical inhibition of the PrL-l/vlPAG circuit decreased the pain threshold in both SNI and sham mice, whereas it showed little effect on anxiety-like behaviors (Figure 3, E–G). Together, these data suggest that the PrL-PAG circuit is vital for regulating pain perception but not anxiety-like behaviors in mice with chronic pain.

### The synaptic activities of PrL^BLA^ and PrL^l/vlPAG^ neurons are differentially altered under chronic pain conditions.

**Figure 4:**
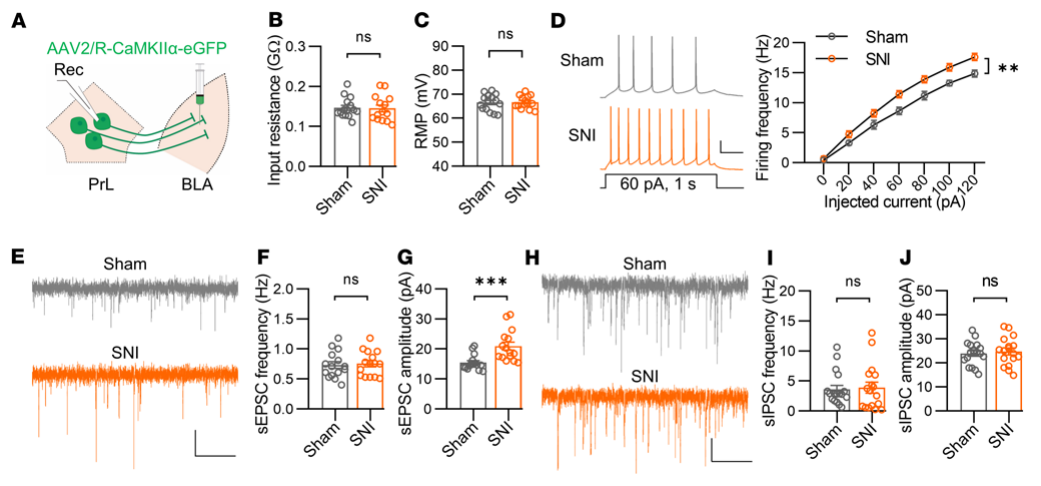
The excitability and excitatory synaptic transmissions of PrL^BLA^ neurons are upregulated under chronic pain conditions. (**A**) Schematic showing viral injection into the BLA and recording of PrL^BLA^ neurons. (**B** and **C**) Comparable input resistance (**B**) and RMP (**C**) of PrL^BLA^ neurons. *n* = 15 and 14 neurons from 4 sham and 4 SNI mice. (**D**) Increased firing frequencies of PrL^BLA^ neurons in SNI mice. Left, representative firing traces. Scale bars, 200 ms, 20 mV. Right, quantitative data. *n* = 25, 21 neurons from 4 sham, 4 SNI mice. (**E**) Representative sEPSC traces. Scale bars: 2 s, 10 pA. (**F** and **G**) Quantifications of sEPSC frequency (**F**) and amplitude (**G**). *n* = 15 neurons from 4 mice per group. (**H**) Representative sIPSC traces. Scale bars: 2 s, 10 pA. (**I** and **J**) Quantifications of sIPSC frequency (**I**) and amplitude (**J**). *n* = 18 and 17 neurons from 4 sham and 4 SNI mice. Data shown as mean ± SEM. **P < 0.01; ***P < 0.001. Mann-Whitney U test (**B**, **G**, and **I**); Student’s *t* test (**C**, **F**, and **J**); 2-way repeated-measures ANOVA (**D**).

To investigate the underlying mechanisms that mediate the differential changes in PrL neuronal activity in chronic pain, we performed whole-cell electrophysiological recording. While the input resistance and resting membrane potential (RMP) in PrL^BLA^ neurons were not different between sham and SNI groups, the firing frequencies in response to injected step-increasing currents were markedly increased in SNI mice compared with sham mice (Figure 4, A–D). Notably, blockade of synaptic activity with antagonists of AMPA, NMDA, and GABAa receptors diminished the differences in firing frequencies between the 2 groups (Supplemental Figure 7A), indicating that altered synaptic activity is responsible for the increased firing of PrL^BLA^ neurons in SNI mice. We further found that the amplitude rather than the frequency of sEPSCs of PrL^BLA^ neurons was dramatically elevated in SNI mice compared with those in sham mice (Figure 4, E–G). In contrast, neither the frequency nor the amplitude of sIPSCs was changed (Figure 4, H–J). These results indicate that excitatory synaptic strength in PrL^BLA^ neurons was increased in mice with chronic pain, probably due to a postsynaptic mechanism.

**Figure 5:**
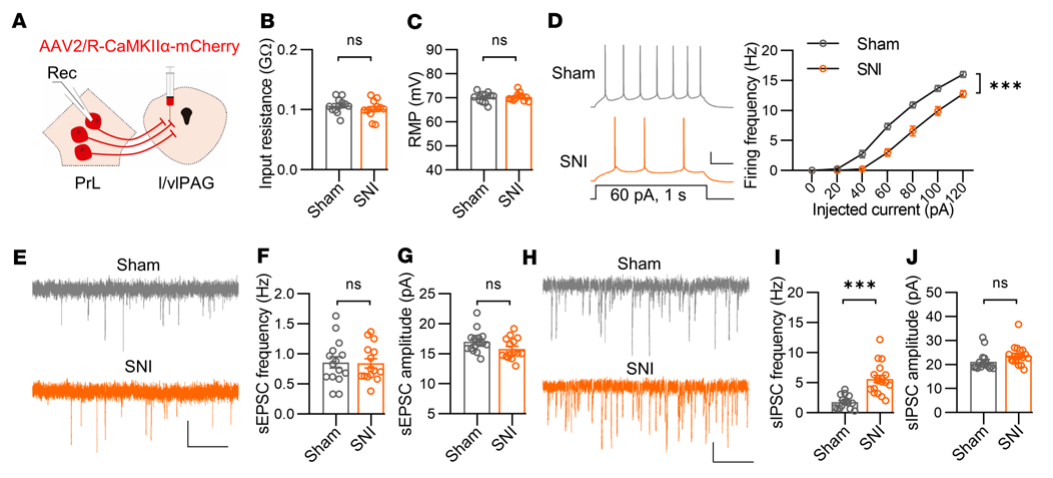
The excitability and inhibitory synaptic transmission of PrL^l/vlPAG^ neurons are down- and upregulated, respectively, under chronic pain conditions. (**A**) Schematic of viral injection into the l/vlPAG and recording of PrL^l/vlPAG^ neurons. (**B** and **C**) Comparable input resistance (**B**) and RMP (**C**) of PrL^l/vlPAG^ neurons. *n* = 12 neurons from 4 mice per group. (**D**) Decreased firing frequencies of PrL^l/vlPAG^ neurons in SNI mice. Left, representative firing traces. Scale bars: 200 ms, 20 mV. Right, quantitative data. *n* = 12 neurons from 4 mice per group. (**E**) Representative sEPSC traces. Scale bars: 2 s, 10 pA. (**F** and **G**) Quantitative data of sEPSC frequency (**F**) and amplitude (**G**). *n* = 15 neurons from 4 mice per group. (**H**) Representative sIPSC traces. Scale bars: 2 s, 10 pA. (**I** and **J**) Quantitative data of sIPSC frequency (**I**) and amplitude (**J**). *n* = 16 and 18 neurons from 4 sham and 4 SNI mice. Data shown as mean ± SEM. ***P < 0.001. Student’s *t* test (**B**, **C**, **F**, **G**, **I**, and **J**); 2-way repeated-measures ANOVA (**D**).

With regard to PrL^l/vlPAG^ neurons, their firing frequencies were significantly decreased in SNI mice compared with those in sham mice, with little changes in input resistance or RMP (Figure 5, A–D). Subsequent analysis of synaptic activities indicated that, while neither the frequency nor the amplitude of sEPSCs was different, the frequency, rather than amplitude, of sIPSCs was increased in SNI mice compared with sham mice (Figure 5, E–J). Altogether, these observations suggest differential changes in the synaptic activity between PrL^BLA^ and PrL^l/vlPAG^ neurons in mice with chronic pain.

### Blockade of postsynaptic GluA1 insertion into PrL^BLA^ neurons alleviates chronic pain–associated anxiety.

**Figure 6:**
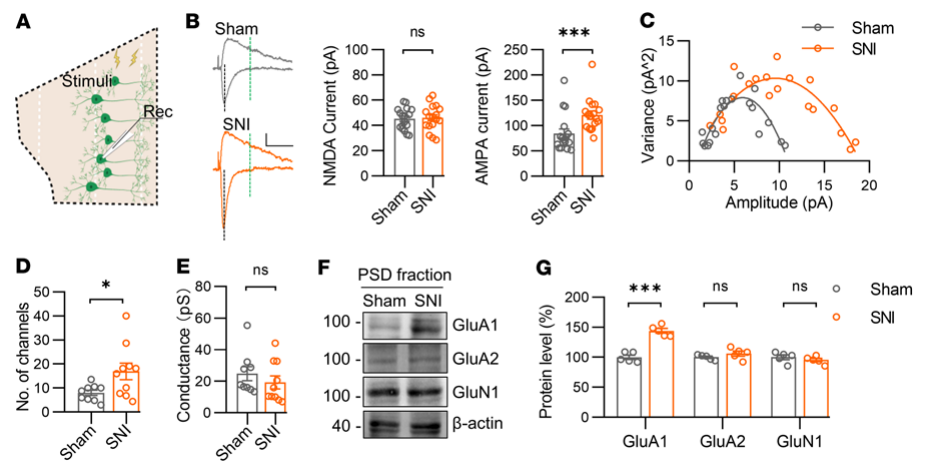
The level of postsynaptic GluA1 is elevated in the PrL of mice with chronic pain. (**A**) Schematic for recording. Rec, record. (**B**) Increased amplitude of AMPAR- but not NMDAR-mediated currents in PrL^BLA^ neurons of SNI mice. Left, representative eEPSC traces. Scale bars: 50 ms and 20 pA. Middle and right, quantification of NMDAR and AMPAR-mediated currents. *n* = 17 neurons from 4 mice per group. (**C**) Representative plot of the variance of sEPSCs amplitudes during decay phase against the mean amplitudes of sEPSCs during decay phase. (**D**) Increased number of channels in PrL^BLA^ neurons of SNI mice. *n* = 9 and 10 neurons from 3 sham and 3 SNI mice. (**E**) Unaltered channel conductance in PrL^BLA^ neurons of SNI mice. *n* = 9 and 10 neurons from 3 sham and 3 SNI mice. (**F**) Representative Western blots. β-actin served as a loading control. (**G**) Quantitation of data in **F**. *n* = 5 mice for each group. Data shown as mean ± SEM. *P < 0.05; ***P < 0.001. Student’s *t* test (**B**, **D**, and **G**); Mann-Whitney U test (**B** and **E**).

Our findings of increased inhibitory activity in PrL^l/vlPAG^ neurons of mice with chronic pain are in line with a previous study reporting a role of cannabinoid receptor 1 (CB1) on parvalbumin-positive (PV-positive) interneurons in enhancing inhibitory activity in PrL^l/vlPAG^ neurons under chronic pain conditions (12). However, the underlying mechanisms through which chronic pain induces increased excitatory synaptic activity in PrL^BLA^ neurons are not known. We found a specific increase in the amplitude of AMPA receptor-mediated (AMPAR-mediated) but not NMDA receptor-mediated (NMDAR-mediated) currents in SNI mice when compared with those in sham controls (Figure 6, A and B). In addition, the presynaptic release probability of glutamate was unchanged as comparable paired-pulse ratios were observed between the 2 groups (Supplemental Figure 7B). These results suggest an augmented function of AMPARs in PrL^BLA^ neurons during chronic pain conditions. To further assess whether chronic pain affects the conductance or number of AMPARs in PrL^BLA^ neurons, we performed an analysis of peak-scaled nonstationary fluctuations of sEPSCs. The results indicate that, while the single-channel conductance was not affected in SNI mice, the number of channels was increased (Figure 6, C–E). These electrophysiological findings were confirmed by a fraction-specific immunoblotting analysis showing that the level of GluA1, but not GluA2 or NMDAR subunits, was increased in the postsynaptic region in the PrL of SNI mice compared with that in sham controls (Figure 6, F and G and Supplemental Figure 7, C and D). Collectively, these results suggest that increased synaptic GluA1 levels in PrL^BLA^ neurons may be responsible for the enhanced neuronal activity and consequent anxiety-like symptoms in SNI mice.

**Figure 7:**
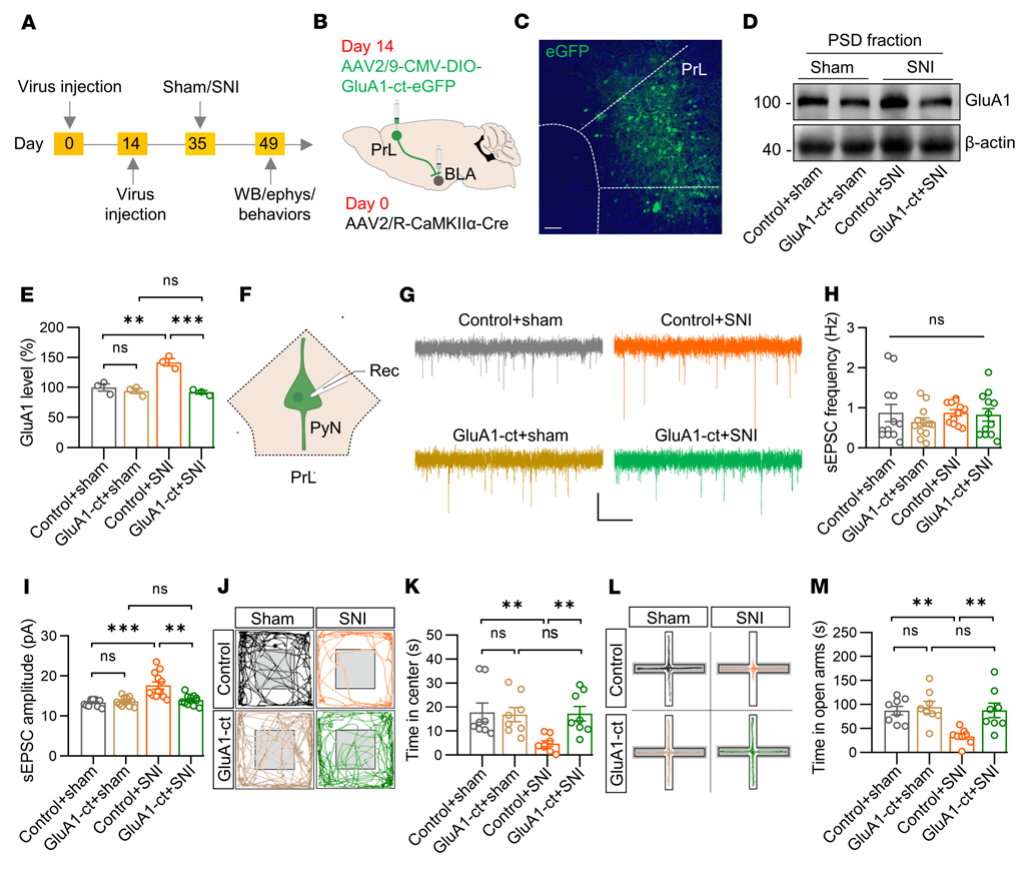
Blockade of postsynaptic GluA1 insertion into PrL^BLA^ neurons alleviates chronic pain-related anxiety. (**A**) Time scheme for experiments. WB, western blotting; ephys, electrophysiological recording. (**B**) Schematic of viral injections. (**C**) Representative image. Scale bar: 50 μm. (**D**) Representative Western blots. β-actin served as a loading control. (**E**) Quantitative data in **D**. *n* = 3 mice per group. (**F**) Schematic of electrophysiological recordings of PrL^BLA^ neurons. (**G**) Representative sEPSC traces. Scale bars: 2 s and 10 pA. (**H**) Unchanged sEPSC frequency by overexpression of GluA1-ct. *n* = 12 neurons from 3 mice per group. (**I**) Reversed sEPSC amplitude in PrL^BLA^ neurons by overexpression of GluA1-ct. *n* = 12 neurons from 3 mice per group. (**J**) Representative traces of mouse travel in OFT. (**K**) Reversed time in center in SNI mice overexpressing GluA1-ct in PrL^BLA^ neurons. *n* = 8 mice per group. (**L**) Representative traces of mice travel in EPM. (**M**) Reversed time in open arms in SNI mice overexpressing GluA1-ct in PrL^BLA^ neurons. *n* = 8 mice per group. Data shown as mean ± SEM. **P < 0.01; ***P < 0.001. 1-way ANOVA followed by posthoc Tukey’s test (**E** and **M**); Kruskal-Wallis test followed by posthoc Dunn’s test (**H**, **I**, and **K**).

To directly test this hypothesis, we attempted to attenuate GluA1 insertion into the postsynaptic membrane of PrL^BLA^ neurons by expressing the C-terminal 81 amino acids of GluA1 (GluA1-ct), which have been reported to block GluA1 insertion into the postsynaptic membrane during plasticity (30, 31). Toward this end, we injected retrograde AAV2/R carrying Cre recombinase into the BLA and an AAV carrying Cre-dependent GluA1-ct tagged with eGFP (AAV2/9-CMV-DIO-GluA1-ct-eGFP) into the PrL (Figure 7, A–C). After 3 weeks, we found that the elevation of postsynaptic GluA1 in SNI mice was diminished by the expression of the GluA1-ct (Figure 7, D and E). Concomitantly, sEPSC amplitude and firing frequencies in PrL^BLA^ neurons of SNI mice were recovered to a level comparable with that in sham mice (Figure 7, F–I and Supplemental Figure 7, E–H). Remarkably, while SNI mice showed higher anxiety levels than sham mice, the blockade of GluA1 insertion ameliorated the anxiety symptoms in SNI mice (Figure 7, J–M and Supplemental Figure 7, I and J). Together, these data demonstrate that postsynaptic GluA1 insertion into PrL^BLA^ neurons was required for enhanced neuronal activity and anxiety symptoms in mice with chronic pain.

### TNF-α promotes the excitatory synaptic activity of PrL^BLA^ neurons and induces anxiety-like behaviors.

**Figure 8:**
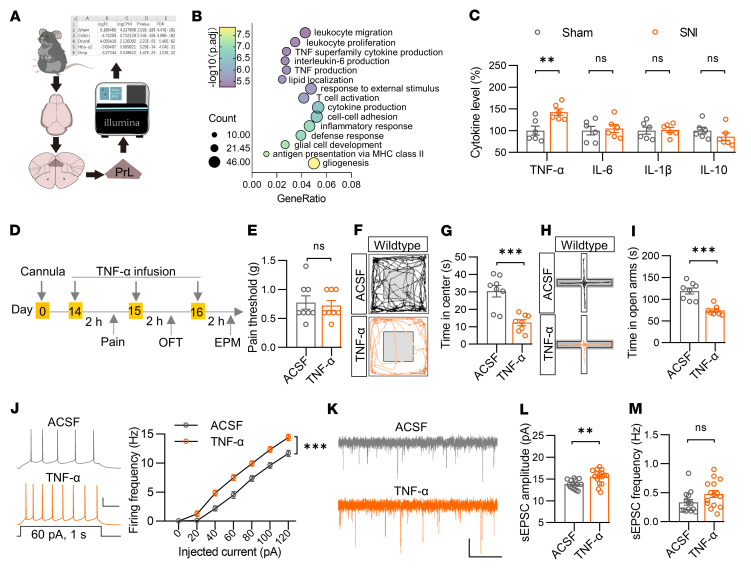
TNF-α promotes the excitatory synaptic activity of PrL^BLA^ neurons and induces anxiety-like behaviors. (**A**) Schematic for experiments. *n* = 4 mice per group. (**B**) Bubble plot of upregulated genes in GO analysis. (**C**) Elevated TNF-α levels in the PrL of SNI mice. *n* = 6 mice per group. (**D**) Time scheme for experiments. (**E**) Unchanged pain threshold in mice with TNF-α injection into the PrL. *n* = 8 mice per group. (**F**) Representative traces of mice travel in OFT. (**G**) Decreased time in center in OFT in mice with TNF-α injection into the PrL. *n* = 8 mice per group. (**H**) Representative traces of mouse travel in EPM. (**I**) Decreased time in open arms in EPM in mice with TNF-α injection into the PrL. *n* = 8 mice per group. (**J**) Increased firing frequencies of PrL^BLA^ neurons. Left, representative firing traces. Scale bars: 200 ms, 20 mV. Right, quantitative data. *n* = 12 neurons from 3 mice per group. (**K**) Representative sEPSC traces. Scale bars, 2 s and 10 pA. (**L** and **M**) Increased sEPSC amplitude (**L**)but not changed frequency (**M**) of PrL^BLA^ neurons in mice with TNF-α injection into PrL. *n* = 14 and 15 neurons from 3 mice for vehicle and TNF-α group, respectively. Data shown as mean ± SEM. **P < 0.01; ***P < 0.001. Student’s *t* test (**C**, **G**, **I**, **L**, and **M**); Mann-Whitney U test (**E**); 2-way repeated measures ANOVA (**J**).

To further determine the underlying mechanisms mediating the elevated excitatory synaptic activity and anxiety symptoms in the chronic pain model, we collected PrL tissues from SNI and sham mice and subjected them to transcriptome analysis (Figure 8A). A total 675 and 921 genes were found to be downregulated and upregulated, respectively, in SNI mice compared with sham control mice (Supplemental Figure 8, A–C). Gene Ontology (GO) analysis indicated that downregulated genes were predominantly related to synaptic functions, such as synaptic assembly, transmission, and plasticity (Supplemental Figure 8D). Intriguingly, the upregulated genes were tightly linked to inflammatory signaling pathways, including leukocyte proliferation, tumor necrosis factor alpha (TNF-α), and IL-6 production (Figure 8B). Direct cytokine analysis in the PrL region showed elevated levels of TNF-α but not IL-6, IL-1β, or IL-10 levels in SNI mice compared with sham mice (Figure 8C). Notably, the TNF-α levels were comparable between the 2 groups 1 week after surgery, and the increase in TNF-α level was preserved at 3 weeks (Supplemental Figure 8E). In addition, female SNI mice, which exhibited hyperalgesia and anxiety-like behaviors (32, 33), also showed increased TNF-α levels in the PrL than the sham controls (Supplemental Figure 9). Together, these observations suggest a common role of TNF-α in chronic pain in mice of both sexes.

To examine whether TNF-α in the PrL plays a role in pain perception or anxiety-like behaviors, we injected recombinant TNF-α protein directly into the PrL region of WT mice (Figure 8D). Note that artificial cerebrospinal fluid (ACSF), but not an unchanged cytokine or BSA, was used as control, because the nonphysiological components may cause artificial gain-of-function effects or inflammatory responses. We found that TNF-α injection failed to change the pain threshold of WT mice (Figure 8E), suggesting little effect on pain perception under the physiological state. However, mice with TNF-α injection spent less time in the center in the OFT than those with ACSF injection (Figure 8, F and G). Moreover, the time in the open arms was decreased by TNF-α injection (Figure 8, H and I). These observations suggest that TNF-α was sufficient to induce anxiety-like behaviors. Electrophysiological analysis showed that TNF-α treatment increased the firing frequencies of PrL^BLA^ neurons and sEPSC amplitude without affecting input resistance, RMP, or sEPSC frequency (Figure 8, J–M and Supplemental Figure 8, F–H). In contrast, neither the firing frequencies nor the amplitude and frequency of sEPSCs in PrL^l/vlPAG^ neurons was changed by TNF-α treatment (Supplemental Figure 10), suggesting a minimal role of TNF-α in the regulation of neuronal and synaptic activities in PrL^PAG^ neurons. Collectively, these results imply that TNF-α specifically participates in the development of anxiety-like behaviors, probably through increasing excitatory synaptic activity and consequent neuronal activity of PrL^BLA^ neurons.

### Blockade of TNF-α signaling in the PrL reverses chronic pain-related anxiety-like behaviors and synaptic deficits.

**Figure 9:**
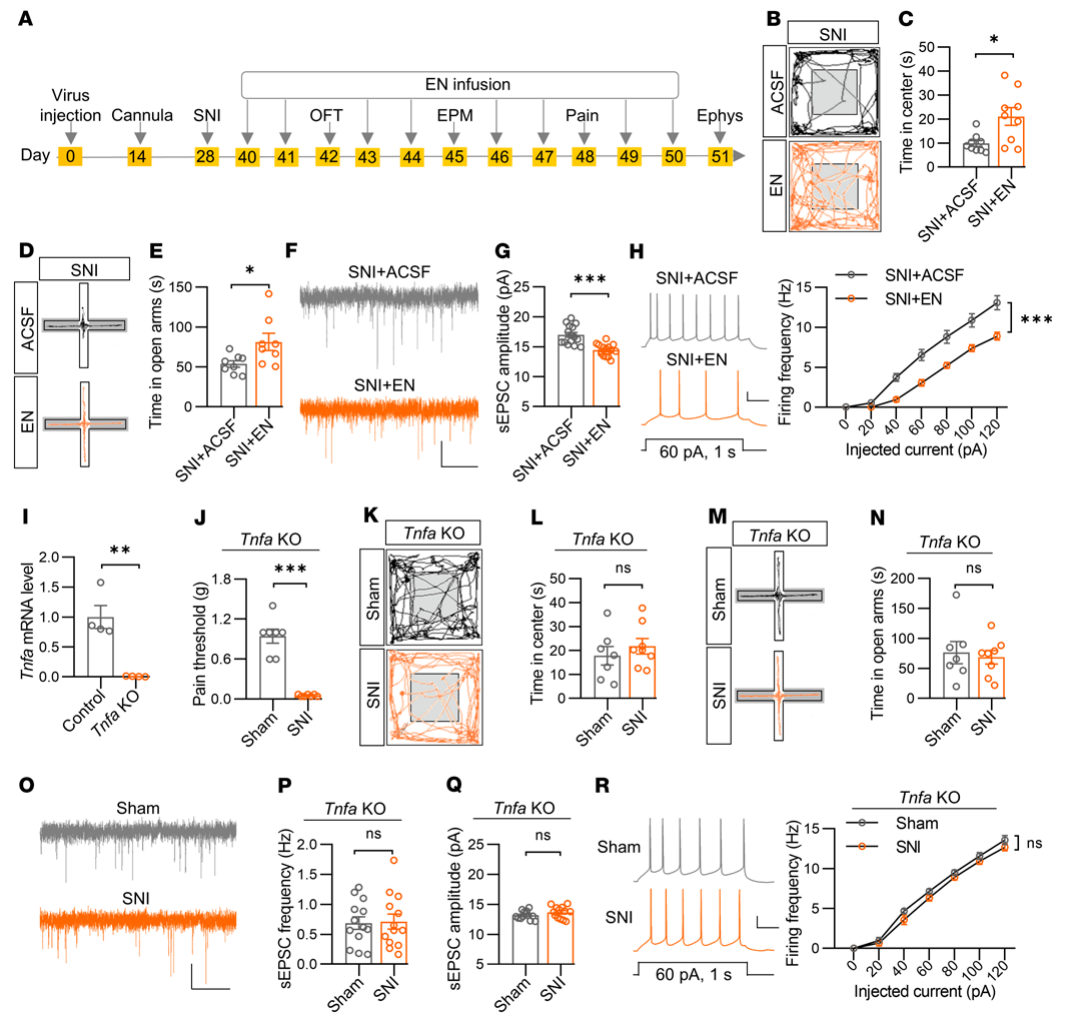
Blockade of TNF-α signaling in the PrL reverses chronic pain-related anxiety-like behaviors and synaptic deficits. (**A**) Time scheme for experiments. (**B** and **D**) Representative traces of mouse travel in OFT (**B**) and EPM (**D**). (**C**) Increased time in center in SNI mice with EN infusion. *n* = 9 mice per group. (**E**) Increased time in open arms in SNI mice with EN infusion. *n* = 8 mice per group. (**F**) Representative sEPSC traces. Scale bar: 2 s, 10 pA. (**G**) Decreased sEPSC amplitude. *n* = 15 neurons from 4 mice per group. (**H**) Reduced firing frequencies. Left, representative firing traces. Scale bars: 200 ms, 20 mV. Right, quantitative data. *n* = 15 and 18 neurons from 4 mice for ACSF and EN group, respectively. (**I**) Undetectable *Tnfa* mRNA levels. *n* = 4 mice per genotype. (**J**) Decreased pain threshold. *n* = 7 mice per group. (**K** and **M**) Representative traces of mouse travel in OFT (**K** and **M**). (**L**) No differences in time in center. *n* = 7 and 8 mice for Sham and SNI groups, respectively. (**N**) No differences in time in open arms. *n* = 7 and 8 mice for Sham and SNI groups, respectively. (**O**) Representative sEPSC traces. Scale bars: 2 s, 10 pA. (**P**) Unchanged sEPSC frequency (**P**) and amplitude (**Q**) of PrL^BLA^ neurons. *n* = 13 neurons from 3 mice per group. (**R**) Unchanged firing frequencies. Left, representative firing traces. Scale bars: 200 ms, 20 mV. Right, quantitative data. *n* = 12 neurons from 3 mice per group. Data shown as mean ± SEM. *P < 0.05; **P < 0.01; ***P < 0.001. Student’s *t* test (**C**, **E**, **G**, **I**, **L**, **N**, **P**, and **Q**); Mann-Whitney U test (**J**); 2-way repeated measures ANOVA (**H** and **R**).

To determine whether TNF-α is required for chronic pain phenotypes in mice, we attempted to block TNF-α signaling by injecting a TNF-α antagonist, Etanercept (EN), into the PrL after SNI surgery (Figure 9A). SNI mice injected with EN showed increased time in the center in the OFT, time in the open arms, and the probability of open-arm entry in the EPM compared with SNI mice injected with ACSF (Figure 9, B–E and Supplemental Figure 11, A and B). However, the pain threshold was not different between the 2 groups (Supplemental Figure 11C). These results suggest that TNF-α in the PrL was required for anxiety-like symptoms but not for hyperalgesia in mice with chronic pain. Furthermore, sEPSC amplitude rather than frequency was decreased in PrL^BLA^ neurons with EN treatment (Figure 9, F and G and Supplemental Figure 11D). Concomitantly, the firing frequencies were reduced (Figure 9H).

To further confirm the role of TNF-α in the regulation of anxiety-like behaviors and synaptic activity, we generated *Tnfa* null (*Tnfa* KO) mice. RT-PCR analysis confirmed that the *Tnfa* mRNA was hardly detectable in *Tnfa* KO mice (Figure 9I). We found that the *Tnfa* KO mice exhibited comparable anxiety levels to their littermates under normal conditions (Supplemental Figure 12, A–F). However, SNI treatment failed to induce anxiety-like behaviors while maintaining a reduced pain threshold in both male and female *Tnfa* KO mice (Figure 9, J–N and Supplemental Figure 12, G–L). Note that the effects of TNF-α on anxiety-like behaviors were not ubiquitous, as *Tnfa* KO mice exhibited significant anxiety-like behaviors induced by chronic restraint stress (CRS) (Supplemental Figure 12, M-R), a well-established stress model of anxiety (19, 34). Electrophysiological analysis showed comparable amplitude and firing frequencies of sEPSCs in PrL^BLA^ neurons between sham and SNI male mice with a *Tnfa* deletion (Figure 9, O–R). As a control, neither the pain threshold nor the anxiety-like behaviors were changed in *IL-6*–deleted mice (Supplemental Figure 13). Together, these data suggest that TNF-α was indispensable in regulating anxiety-like behaviors in mice with chronic pain through the upregulation of synaptic activity of PrL^BLA^ neurons.

### TNFR1 in PrL^BLA^ neurons is required for dysfunction of excitatory synaptic activity and anxiety-like behaviors in chronic pain.

**Figure 10:**
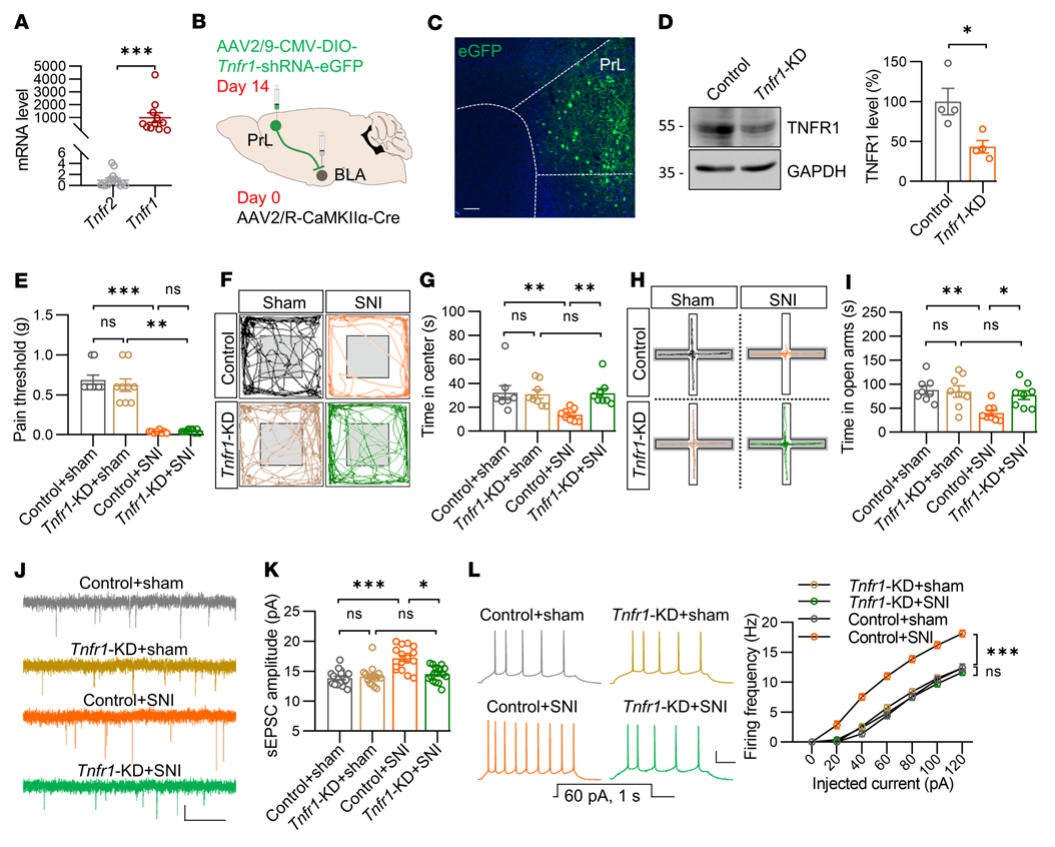
TNFR1 in PrL^BLA^ neurons is required for dysfunction of excitatory synaptic activity and anxiety-like behaviors in chronic pain. (**A**) Higher level of *Tnfr1* in PrL^BLA^ neurons. *n* = 14 and 11 tubes for *Tnfr2* and *Tnfr1*. (**B**) Schematic for viral injections. (**C**) Representative image; dotted lines denote the boundaries of PrL. Scale bar: 50 μm. (**D**) Left, representative Western blots. GAPDH served as a loading control. Right, quantified data. *n* = 4 mice per group. (**E**) Unaltered pain threshold in *Tnfr1*-KD mice. *n* = 9, 9, 8, and 9 mice for Control+sham, *Tnfr1*-KD+sham, Control+SNI, and *Tnfr1*-KD+SNI group, respectively. (**F** and **H**) Representative traces of mice travel in OFT (**F**) and in EPM (**H**). (**G**) Increased time in center in SNI mice by deletion of *Tnfr1* in PrL^BLA^ neurons. *n* = 8 mice per group. (**I**) Reversed time in open arms in SNI mice by deletion of *Tnfr1* in PrL^BLA^ neurons. *n* = 8 mice per group. (**J**) Representative sEPSC traces. Scale bars: 2 s and 10 pA. (**K**) Not changed sEPSC amplitudes in SNI mice by deletion of *Tnfr1* in PrL^BLA^ neurons. *n* = 18, 16, 16, and 17 neurons from 4 mice for Control+sham, *Tnfr1*-KD+sham, Control+SNI, and *Tnfr1*-KD+SNI groups, respectively. (**L**) Diminished difference in firing frequencies. Left, representative firing traces. Scale bars: 200 ms, 20 mV. Right, quantitative data. *n* = 15 neurons from 4 mice per group. Data shown as mean ± SEM. *P < 0.05; **P < 0.01; ***P < 0.001. Mann Whitney U test (**A**); Student’s *t* test (**D**); Kruskal-Wallis test followed by Dunn’s test (**E**, **G**, and **K**); 1-way ANOVA followed by posthoc Tukey’s test (**I**); 2-way repeated-measures ANOVA (**L**).

TNF-α functions via binding to its receptors, including TNFR1 and TNFR2 (35). To determine which receptor mediates the effects of TNF-α on excitatory synaptic activity and anxiety-like behaviors under chronic pain conditions, we first determined the expression of these receptors in PrL^BLA^ neurons using single-cell RT-PCR analysis. The level of *Tnfr1* mRNA was significantly higher than that of *Tnfr2* in PrL^BLA^ neurons (Figure 10A). These data imply that TNFR1 is the predominant receptor for TNF-α in the PrL^BLA^ neurons. Transcriptomic analysis indicated comparable *Tnfr1* mRNA levels between sham and SNI groups (*P*_adj_ = 0.357), suggesting that the TNFR1 expression in the PrL was not altered in chronic pain. To test whether TNFR1 mediated the effects of TNF-α on chronic pain-induced anxiety, we knocked down *Tnfr1* specifically in PrL^BLA^ neurons with an injection of retrograde AAV-Cre virus into the BLA and AAV-DIO-*Tnfr1*-shRNA-eGFP into the PrL (Figure 10, B and C). Western blotting analysis indicated that the TNFR1 protein level was decreased in the PrL region (Figure 10D). *Tnfr1* knockdown in PrL^BLA^ neurons of SNI mice increased the time in the center in the OFT and time in the open arms in the EPM to a similar level of those in sham mice but showed little effect on pain threshold (Figure 10, E–I). These observations indicate a specific alleviating effect of *Tnfr1* knockdown in PrL^BLA^ neurons on anxiety-like behaviors in SNI mice. Electrophysiologically, the difference in sEPSC amplitude and firing frequencies of PrL^BLA^ neurons between sham and SNI mice was diminished when the *Tnfr1* was knocked down (Figure 10, J–L). Altogether, these observations demonstrate that TNFR1 in PrL^BLA^ neurons contributed to the dysfunction of excitatory synaptic activity and anxiety-like behaviors in chronic pain.

## Discussion

Chronic pain can be accompanied by negative emotions such as anxiety (1, 2, 4, 36). We believe that the present study provides compelling evidence demonstrating that maladaptation of distinct top-down circuits from the PrL underlies hyperalgesia and anxiety-like behaviors, respectively, under chronic pain conditions. Further, we report a critical role of TNF-α signaling in the PrL in mediating chronic pain–induced anxiety-like behaviors. We believe that these results reveal novel circuitry and molecular mechanisms for the pathogenesis of chronic pain and provide potential targets for therapeutic interventions.

Both human and rodent studies have demonstrated a critical role of mPFC in pain signaling processing and affective modulation during acute and chronic pain (11, 36). Notably, clinical studies have reported that noninvasive stimulation of the dorsolateral PFC (the homolog of mPFC in rodents) in humans could effectively alleviate hyperalgesia symptoms (37, 38). However, how functional integration of nociceptive information and affection within the mPFC network change at the circuit and molecular levels during pain chronicity remains elusive. Previous work has shown that pyramidal neurons in PrL layer 2/3 and layer 5 display increased and reduced excitability, respectively, in a mouse model of neuropathic pain (20, 39, 40). These findings suggest a layer-specific change in neuronal activity under chronic pain conditions. However, the evidence regarding how it occurs at the circuit level and whether this phenomenon exists in vivo is lacking. These data provide a circuit-level resolution using viral tracing and in-vivo 2P Ca^2+^ imaging techniques, showing that PrL pyramidal neurons in the PrL-BLA circuit are primarily located in layer 2/3 and display higher activity during chronic pain. In contrast, PrL pyramidal neurons in the PrL-l/vlPAG circuit are mainly found in layer 5 and are hypoactive in vivo. These results extend previous findings and afford a frame to dissect their roles in pain perception and related anxiety-like behaviors under chronic pain conditions. It is of note that, while the phenotype of reduced activity in mPFC layer 5 under chronic pain has been reported in several studies (12, 20), 1 study hinted decreased rather than increased excitability of PrL neurons in layer 2/3 in a model of inflammatory pain (9). These discrepancies in the results may be due to the use of different animal models of chronic pain and raise the possibility of a PrL-BLA circuit–specific change in neuronal activity in PrL layer 2/3 during pain chronicity.

Pain involves both nociceptive information and affective processing. Whether and how convergent or divergent pathways mediate these distinct components are unclear. Using optogenetic manipulations, we demonstrate that the PrL-BLA and PrL-l/vlPAG circuits individually mediate anxiety-like behaviors and hyperalgesia in chronic pain, offering what we believe to be a new line of evidence to support the divergent-pathway-centered hypothesis. Elevated activity of the PrL-BLA circuit has been reported in various studies using different stress models, including chronic restraint stress, acute social defeat stress, and chronic ethanol exposure and withdrawal (19, 41, 42); it appears that adaptation of this circuit serves as a general mechanism for the development of anxiety-like behaviors. It is noteworthy that optogenetic activation of the PrL-BLA circuit in early SNI mice but not in sham mice significantly induced anxiety-like behaviors, suggesting that the effect of the PrL-BLA circuit on anxiety-like behaviors may be context dependent. SNI surgery may serve as a trigger for inducing anxiety-like behaviors, which could be promoted by the activation of the PrL-BLA circuit.

l/vlPAG is the key node for descending pain modulation(43). Increased activity in l/vlPAG is expected to compromise ascending nociceptive processing through the activation of the rostroventral medulla (RVM) and Locus coeruleus (LC), thus causing analgesic effects (11). Our results are consistent with previous reports showing that excitatory neurons in the PrL send top-down afferent input into the l/vlPAG, and that modulation of PrL-l/vlPAG circuit activity affects pain perception (10, 12). A recent paper reported that activation of the dorsal mPFC-vlPAG neural pathway produces both analgesic and anxiolytic effects in mice using a common peroneal nerve ligation (CPNL), another model for chronic neuropathic pain (10). However, we found that stimulation of the PrL neuronal terminal in the l/vlPAG only induced an analgesic effect without influencing anxiety-like behaviors. While the exact reasons for these discrepancies are unclear, we speculate that it may be due to differences in mouse models, specifically CPNL versus SNI, and target regions in these studies. Rostral anterior cingulate cortex (rACC) and PrL constitute dorsal mPFC. ACC has been shown to be essential in regulating pain and related emotional deficits under chronic pain conditions (2, 44). It is possible that the rACC-l/vlPAG pathway works in a complementary manner to modulate pain-related anxiety-like behaviors. Nevertheless, future investigations are warranted to dissect the roles of distinct circuits from different subregions of the mPFC in governing chronic pain and comorbid emotional dysfunctions.

Previous work reported that GABAergic feed-forward inhibition by PV-positive interneurons — probably mediated by presynaptic endocannabinoid signaling and metabotropic glutamate receptor 1 — is enhanced in the PrL region in a model of neuropathic pain, which leads to the deactivation of PrL-l/vlPAG pathway and consequent pain hypersensitivity (7, 10, 12, 20). Our in-vivo 2P Ca^2+^ imaging study on live mice affords strong evidence demonstrating its occurrence in vivo by showing that the spontaneous activity of PrL^l/vlPAG^ neurons was decreased after SNI surgery. Furthermore, we found increased sIPSC frequency in PrL^l/vlPAG^ neurons, which is in line with the reports of enhanced GABAergic tone in the PrL region under chronic pain conditions. Conversely, we found increased amplitude but not frequency of sEPSCs, with little change in sIPSCs, in PrL^BLA^ neurons of SNI mice. These findings were further proved by the result showing increased synaptic GluA1 expression in the PrL of SNI mice. Considering the unaltered intrinsic excitability of PrL^BLA^ neurons, it is most likely that increased excitatory input accounts for the elevated activity of PrL^BLA^ neurons during chronic pain. This notion is further strengthened by the observations that the blockade of GluA1 insertion into the postsynaptic membrane of PrL^BLA^ neurons alleviates comorbid anxiety-like behaviors in mice with chronic pain. Notably, the increase in synaptic GluA1 expression in the PrL of SNI mice is rather dramatic (with a mean of 144.7%). Because the PrL^BLA^ neurons comprise a minor proportion in the PrL, and the PrL connects with various other brain regions, including the nucleus accumbens (NAc) and ventral tegmental area (VTA) (6), it is reasonable to speculate that increased GluA1 expression may also occur in the PrL neurons projecting to other regions in SNI mice. Moreover, whether it occurs in the GABAergic inhibitory neurons, which constitute approximately 20% of the total population in the mPFC (45), awaits further investigation in future studies.

Chronic pain is widely known to be associated with chronic neuroinflammation both in the peripheral and central nervous systems (46, 47). During chronic neuroinflammation, multiple proinflammatory mediators such as TNF-α and IL-1β are released from distinct cell types and play an important role in promoting pain sensation and related affective abnormalities (48). Besides its role in the inflammatory response, TNF-α also exerts homeostatic functions, such as the regulation of synaptic plasticity, by triggering the surface expression of glutamate receptors (49). We provide multiple lines of evidence demonstrating that TNF-α in the PrL served as a trigger for elevated PrL-BLA circuit activity; this eventually was the cause of chronic pain–induced anxiety-like behaviors. Firstly, TNF-α signaling and its protein level were increased in the PrL, and TNF-α expression positively correlated with anxiety level in both sham and SNI mice. Secondly, direct infusion of TNF-α into the PrL induced anxiety-like behaviors without influencing the pain threshold. Thirdly, both blockade of TNF-α signaling with an antagonist and genetic deletion of *Tnfa* attenuated the anxiety-like behaviors in chronic pain. Lastly, specific knockdown of *Tnfr1* in the PrL alleviated the anxiety-like behaviors in mice with chronic pain. All of these results point to a fundamental role of TNF-α signaling in the PrL in mediating anxiety-like behaviors during chronic pain. It is worth noting that the effects of TNF-α in the PrL are rather circuit-specific, as TNF-α minimally affects the synaptic activity in PrL^l/vlPAG^ neurons and thus displays little impact on pain perception. We speculate that it may be due to the lower expression levels of TNF-α receptors and/or downstream signaling molecules in the PrL^l/vlPAG^ neurons. Furthermore, it is known that various cell types, including microglia, astrocytes, and peripheral inflammatory cells, secrete TNF-α (49), however, the cell type(s) releasing TNF-α in the PrL under chronic pain conditions remain unknown. Future investigations are required to define the contribution of TNF-α derived from distinct PrL cell types (individually or synergistically) to chronic pain-related anxiety-like behaviors.

Accumulated anatomical evidence has demonstrated a reciprocal connection between PrL and BLA (6, 19). Notably, a previous study reports a critical role of BLA-PrL projections in pain hypersensitivity after peripheral nerve injury (12). Based on our findings, which show a crucial role of PrL-BLA projections in anxiety-like behaviors but not in pain perception, we hypothesized that peripheral nerve injury initially elevates the activity of BLA neurons projecting to PrL GABAergic interneurons, which cause inactivation of PrL^l/vlPAG^ neurons and lead to hyperalgesia. On the other hand, TNF-α accumulates in the PrL and further promotes the activity of BLA neurons by targeting PrL^BLA^ neurons, which causes anxiety phenotypes and may further disturb pain perception. This feedback loop, being a vicious cycle, probably sets the foundation for the difficulty in clinical treatment of chronic pain. Further investigations are needed to validate this hypothesis. Nevertheless, our study offers a potential intervention strategy for chronic pain with comorbid anxiety.

## Methods

Detailed methods are provided in the Supplemental Material.

### Statistics.

Adult male and female mice of at least 8 weeks of age were used in the present study. Animal or cell numbers for each experiment and statistical methods are described in the figure legends, and results of the statistical analyses, such as degrees of freedom and exact *P* values, are described in Supplemental Materials. Statistical analyses were performed using GraphPad Prism. The sample size choice was made based on previous studies (50, 51), not predetermined by a statistical method. The Shapiro-Wilk test was used to evaluate the normality of the data distribution before any further analysis. When data was normally distributed, a 2-tailed Student’s *t* test was used to compare data from 2 independent groups; regular or repeated measures 1-way ANOVA with Tukey’s posthoc test was used to compare data from more than 2 groups; 2-way repeated measures ANOVA with posthoc Šidák’s test was used for the investigation of behaviors in chronic pain model at different time points and firing frequency analysis. When data were not normally distributed, the Mann-Whitney U test was used to compare data from 2 independent groups; Kruskal-Wallis test or Friedman’s test followed by Dunn’s posthoc test was used to compare data from more than 2 groups. All tests were 2-sided except that the 1-sided Mann-Whitney U test was used to identify the responsive neurons in 2P Ca^2+^ imaging experiments. Data represent mean ± SEM. *P* < 0.05 was considered statistically significant.

### Study approval.

These studies were approved by the Animal Ethics Committee of Guangzhou Medical University.

## Author contributions

XDS, FG, JH, and QLY designed the study. FG, JH, GBH, QLY, SY, STZ, JL, CHW, GFC, SML, YLZ, and ZY performed research. FG, JH, GBH, QLY, and SY analyzed data with supervision from YPN, SL, and BJH.XDS and FG wrote the manuscript. The order of co–first authors was based on the order with which the individuals joined the project.

## Supplementary Material

Supplemental data

Supplemental video 1

Supplemental video 2

Supplemental video 3

Supplemental video 4

## Version 1 03/14/2023

In-Press Preview

## Version 2 05/01/2023

Electronic publication

## References

[1] Cohen SP (2021). Chronic pain: an update on burden, best practices, and new advances. Lancet.

[2] Zhuo M (2016). Neural mechanisms underlying anxiety-chronic pain interactions. Trends Neurosci.

[3] Kuner R, Flor H (2016). Structural plasticity and reorganisation in chronic pain. Nat Rev Neurosci.

[4] Baliki MN, Apkarian AV (2015). Nociception, pain, negative moods, and behavior selection. Neuron.

[5] Ong WY (2019). Role of the prefrontal cortex in pain processing. Mol Neurobiol.

[6] Anastasiades PG, Carter AG (2021). Circuit organization of the rodent medial prefrontal cortex. Trends Neurosci.

[7] Zhang Z (2015). Role of prelimbic GABAergic circuits in sensory and emotional aspects of neuropathic pain. Cell Rep.

[8] Ji G (2010). Cognitive impairment in pain through amygdala-driven prefrontal cortical deactivation. J Neurosci.

[9] Wang GQ (2015). Deactivation of excitatory neurons in the prelimbic cortex via Cdk5 promotes pain sensation and anxiety. Nat Commun.

[10] Yin JB (2020). dmPFC-vlPAG projection neurons contribute to pain threshold maintenance and antianxiety behaviors. J Clin Invest.

[11] Tan LL, Kuner R (2021). Neocortical circuits in pain and pain relief. Nat Rev Neurosci.

[12] Huang J (2019). A neuronal circuit for activating descending modulation of neuropathic pain. Nat Neurosci.

[13] Negrón-Oyarzo I (2014). Effects of chronic stress in adolescence on learned fear, anxiety, and synaptic transmission in the rat prelimbic cortex. Behav Brain Res.

[14] Suzuki S (2016). The infralimbic and prelimbic medial prefrontal cortices have differential functions in the expression of anxiety-like behaviors in mice. Behav Brain Res.

[15] Saitoh A (2014). Activation of the prelimbic medial prefrontal cortex induces anxiety-like behaviors via N-Methyl-D-aspartate receptor-mediated glutamatergic neurotransmission in mice. J Neurosci Res.

[16] Lisboa SF (2010). Different role of the ventral medial prefrontal cortex on modulation of innate and associative learned fear. Neuroscience.

[17] Hare BD, Duman RS (2020). Prefrontal cortex circuits in depression and anxiety: contribution of discrete neuronal populations and target regions. Mol Psychiatry.

[18] Janak PH, Tye KM (2015). From circuits to behaviour in the amygdala. Nature.

[19] Liu WZ (2020). Identification of a prefrontal cortex-to-amygdala pathway for chronic stress-induced anxiety. Nat Commun.

[20] Cheriyan J, Sheets PL (2018). Altered excitability and local connectivity of mPFC-PAG neurons in a mouse model of neuropathic pain. J Neurosci.

[21] Anastasiades PG (2019). Cell-type-specific D1 dopamine receptor modulation of projection neurons and interneurons in the prefrontal cortex. Cereb Cortex.

[22] Gabbott PL (2005). Prefrontal cortex in the rat: projections to subcortical autonomic, motor, and limbic centers. J Comp Neurol.

[23] Vertes RP (2004). Differential projections of the infralimbic and prelimbic cortex in the rat. Synapse.

[24] Ho Kim S, Mo Chung J (1992). An experimental model for peripheral neuropathy produced by segmental spinal nerve ligation in the rat. Pain.

[25] Decosterd I, Woolf CJ (2000). Spared nerve injury: an animal model of persistent peripheral neuropathic pain. Pain.

[26] Cho JH (2013). Synaptic encoding of fear extinction in mPFC-amygdala circuits. Neuron.

[27] Dopfel D (2019). Individual variability in behavior and functional networks predicts vulnerability using an animal model of PTSD. Nat Commun.

[28] Tillage RP (2021). Co-released norepinephrine and galanin act on different timescales to promote stress-induced anxiety-like behavior. Neuropsychopharmacology.

[29] Lee JY (2022). Role of anterior cingulate cortex inputs to periaqueductal gray for pain avoidance. Curr Biol.

[30] Li K (2013). βCaMKII in lateral habenula mediates core symptoms of depression. Science.

[31] Rumpel S (2005). Postsynaptic receptor trafficking underlying a form of associative learning. Science.

[32] Mecca CM (2021). Dynamic change of endocannabinoid signaling in the medial prefrontal cortex controls the development of depression after neuropathic pain. J Neurosci.

[33] Sieberg CB (2018). Neuropathic pain drives anxiety behavior in mice, results consistent with anxiety levels in diabetic neuropathy patients. Pain Rep.

[34] Zhang WH (2021). Amygdala circuit substrates for stress adaptation and adversity. Biol Psychiatry.

[35] Fischer R (2020). Selective targeting of TNF receptors as a novel therapeutic approach. Front Cell Dev Biol.

[36] Kuner R, Kuner T (2021). Cellular circuits in the brain and their modulation in acute and chronic pain. Physiol Rev.

[37] Seminowicz DA, Moayedi M (2017). The dorsolateral prefrontal cortex in acute and chronic pain. J Pain.

[38] Taylor JJ (2012). Endogenous opioids mediate left dorsolateral prefrontal cortex rTMS-induced analgesia. Pain.

[39] Mitrić M (2019). Layer- and subregion-specific electrophysiological and morphological changes of the medial prefrontal cortex in a mouse model of neuropathic pain. Sci Rep.

[40] Cordeiro Matos S (2015). Peripheral neuropathy induces HCN channel dysfunction in pyramidal neurons of the medial prefrontal cortex. J Neurosci.

[41] Grossman YS (2022). Structure and function differences in the prelimbic cortex to basolateral amygdala circuit mediate trait vulnerability in a novel model of acute social defeat stress in male mice. Neuropsychopharmacology.

[42] McGinnis MM (2020). Chronic ethanol differentially modulates glutamate release from dorsal and ventral prefrontal cortical inputs onto rat basolateral amygdala principal neurons. eNeuro.

[43] François A (2017). A brainstem-spinal cord inhibitory circuit for mechanical pain modulation by GABA and enkephalins. Neuron.

[44] Li XH (2021). Oxytocin in the anterior cingulate cortex attenuates neuropathic pain and emotional anxiety by inhibiting presynaptic long-term potentiation. Cell Rep.

[45] DeFelipe J (2013). New insights into the classification and nomenclature of cortical GABAergic interneurons. Nat Rev Neurosci.

[46] Ji RR (2016). Pain regulation by non-neuronal cells and inflammation. Science.

[47] Sommer C (2018). Inflammation in the pathophysiology of neuropathic pain. Pain.

[48] Ji RR (2018). Neuroinflammation and central sensitization in chronic and widespread pain. Anesthesiology.

[49] Olmos G, Lladó J (2014). Tumor necrosis factor alpha: a link between neuroinflammation and excitotoxicity. Mediators Inflamm.

[50] Sun XD (2016). Lrp4 in astrocytes modulates glutamatergic transmission. Nat Neurosci.

[51] Sun XD (2018). Neogenin in amygdala for neuronal activity and information processing. J Neurosci.

