# Supplementary material for: Elevated prelimbic cortex-to-basolateral amygdala circuit activity mediates comorbid anxiety-like behaviors associated with chronic pain
Source: J Clin Invest. 2023 May 1;133(9):e166356. doi: 10.1172/JCI166356 (PMC10145931; doi:10.1172/JCI166356)
Supplement: Supplemental data [file jci-133-166356-s116.pdf]

# Supplemental Materials


- **Methods………………………………………………………………………....2**
- **References………………………………………………………………….21**
- **Supplemental Figures………………………………………………………22**
- **Statistical results…………………………………………………………43**

## Methods

### Animals

Eight- to ten-week-old male and female mice were used for experiments. C57BL/6J mice were purchased from Guangdong Medical Laboratory Animal Center. *Tnfa* KO mice (stock#: 005540) and *Il6* KO mice (stock#: 002650) were purchased from Jackson Laboratory. The following primers were used for genotyping: *Tnfa* KO: 5'-TAG CCA GGA GGG AGA ACA GA-3', 5'-AGT GCC TCT TCT GCC AGT TC-3' and 5'-CGT TGG CTA CCC GTG ATA TT-3'; *IL-6* KO, 5'-TTC CAT CCA GTT GCC TTC TTG G-3', 5'-AGT GCC TCT TCT GCC AGT TC-3' and 5'-CCG GAG AAC CTG CGT GCA ATC C-3'. Mice had access to food and water ad libitum and were housed under the condition of 12 hr light/dark cycle (light on at 6 am) with a temperature of 22 ± 1°C and > 30% humidity. Animal experiments were carried out following the Guidelines for Animal Care and Use of China. The animal experimental protocols were approved by the Animal Ethics Committee of Guangzhou Medical University.

### Reagents and antibodies

Chemicals were purchased from Sigma-Aldrich unless otherwise indicated. 6-cyano-7-nitroquinoxaline-2,3-dione (CNQX, 0190), DL-2-amino-5-phosphonopentanoic acid (DL-AP5, 0105), and SR-95531 (1262) were purchased from Tocris Bioscience. Recombinant TNF-a protein was procured from R&D system (#210-TA-020/CF), and Etanercept (EN) was obtained from Pfizer. Following antibodies were used in the present study: rabbit anti-CaMKIIα

(Abcam, ab5683, 1:250 for immunofluorescent staining); rabbit anti-GluA1 (Cell Signaling Technology, #13185, 1:1000 for immunoblotting); rabbit anti-GluA2 (Cell Signaling Technology, #13607, 1:1000 for immunoblotting); rabbit anti-GluN1 (Cell Signaling Technology, #5704S, 1:1000 for immunoblotting); rabbit anti-GluN2A (Cell Signaling Technology, #4205S, 1:1000 for immunoblotting); rabbit anti-GluN2B (Cell Signaling Technology, #14544, 1:1000 for immunoblotting); mouse anti-TNFR1 (Santa Cruz Biotechnology, sc-8436, 1:500 for immunoblotting); mouse anti-β-actin (Sigma-Aldrich, A1978, 1:8000 for immunoblotting); mouse anti-GAPDH (Proteintech, 60004-1, 1:10000 for immunoblotting).

**Chronic pain mouse model**

A neuropathic pain mouse model, as described previously, was utilized as a chronic pain model (1). In brief, mice were anesthetized via inhalation of isoflurane (2-3%). A 1 cm-long incision was made on the skin and muscle of the left thigh, followed by blunt dissection until the three terminal branches of the sciatic nerve (the sural, common peroneal, and tibial nerves) were exposed. Common peroneal and tibial nerves were ligated with 4/0 chromic gut sutures. Approximately 2 mm of fractions were cut off below the suture of nerves while the sural nerve was kept intact. The incision was then sutured with surgical knots and erythromycin ointment was applied. Mice in the sham group had the same surgical procedure except nerve ligation.

**Chronic restraint stress model**

The chronic restraint stress model was generated as described previously (2). Briefly, adult male mice were placed in plastic tubes with holes for airflow for 2 h (1 pm–3 pm) per day for 10 consecutive days. The control mice were placed in the test room for 2h without restraint, and then returned to their home cages. The anxiety-like behaviors of these mice were examined on the day after the last training day.

**Behavioral tests**

All behavioral tests were performed during the light phase (1 pm–5 pm). Mice were kept in the testing room (dim light: ~60 lux) for habituation for at least 1hr before the test. The mice's behaviors were monitored using an overhead camera and tracking software (EthoVision XT, Noldus). Areas were cleaned with 75% ethanol to get rid of olfactory disturbance between tests. The details of behavioral tests are as follows:

**Mechanical pain threshold test**: Von Frey test was utilized to detect the mechanical pain threshold (3). Briefly, mice were placed in a plastic cylinder on an elevated wire mesh table for 30 min for acclimatization. A set of von Frey filaments (0.008-4 g, Stoelting, IL, USA) was used to test the mechanical withdrawal threshold of hindpaws (ipsilateral to the operation). During the test, a single filament was perpendicularly pressed against the lateral plantar surface of hindpaws in ascending order of strength. The gauge of filament was recorded as

the mechanical pain threshold when mice showed nociceptive behaviors such as sudden paw withdrawal, flinching, or paw licking in three out of five stimuli.

**Open field test:** The mouse was gently placed in the center of an open field apparatus (40 × 40 × 40 cm) and was allowed to explore the arena for 5 min. The total distance was analyzed as a measure of locomotor ability, and the time spent in the center of the arena (20 × 20 cm) was processed as an evaluation of the anxiety-like behavior.

**Elevated plus-maze test:** As described previously (4), an elevated plus-maze which consists of two opposing wall-closed arms (30 × 5 × 15 cm), two opposing open arms (30 × 5 × 0.5 cm), and a center platform (5 × 5 cm) was used to create an approach-avoidance conflict environment. Each mouse was rapidly put into the central platform facing an open arm, and movements were recorded in a 5-min session. The time spent in the open arms and the ratio of open-arm entries to total arm entries were analyzed to assess the general anxiety level.

**Virus injection, drug delivery, and fiberoptic cannula implantation**

As described previously (5), mice were anesthetized with isoflurane (2 - 3%) and head-fixed in a stereotaxic device (RWD Life Science. Inc, China). An incision was made through the scalp to expose the skull for drilling. For virus injection, a specific volume of the virus was injected into the BLA (coordinates from bregma: AP, -1.20 mm; DV, –4.75 mm; ML, ±3.20 mm), l/vlPAG (coordinates from bregma: AP, -4.85 mm; DV, –3.00 mm; ML, ±0.35 mm) or PrL (coordinates from bregma:

AP, +2.20 mm; DV, –1.90 mm; ML, ±0.30 mm) through a pulled glass capillary, controlled by a microinjector (Nanoliter 2020, World Precision Instruments) at a rate of 20 nl/min. The capillary was retracted slowly 10 min after the injection. Incision site was sutured and covered with erythromycin ointment. Mice were then returned to their home cages for recovery.

The following AAV vectors were used in the present study: AAV2/R-CaMKIIα-eGFP (titer: 5.24 × $10^{12}$ v.g./ml, 0.1 μl unilateral or bilateral into BLA, BrainVTA); AAV2/R-CaMKIIα-mCherry (titer: 5.00 × $10^{12}$ v.g./ml, 0.2 μl unilateral into l/vlPAG, BrainVTA); AAV2/R-CaMKIIα-GCaMP6s (titre: 5.06 × $10^{12}$ v.g./ml, 0.2 μl unilateral into BLA or l/vlPAG, BrainVTA); AAV2/9-CaMKIIα-ChR2-mCherry (titer: 3.74 × $10^{12}$ v.g./ml, 0.2 μl bilateral into PrL, BrainVTA); AAV2/9-CaMKIIα-eNpHR-mCherry (titer: 2.93 × $10^{12}$ v.g./ml, 0.2 μl bilateral into PrL, BrainVTA); AAV2/9-CaMKIIα-mCherry (titer: 5.14 × $10^{12}$ v.g./ml, 0.2 μl bilateral into PrL, BrainVTA); AAV2/9-CaMKIIα-Cre (titer: 5.85 × $10^{12}$ v.g./ml, 0.2 μl bilateral into BLA, BrainVTA); AAV2/9-CMV-DIO-GluA1-ct-eGFP (titer: 2.22 × $10^{13}$ v.g./ml, 0.2 μl bilateral into PrL); AAV2/9-CMV-DIO-eGFP (titer: 9.16 × $10^{12}$ v.g./ml, 0.2 μl bilateral into PrL); AAV2/9-CMV-DIO-*Tnfr1*-shRNA-eGFP (titer: 5.18 × $10^{12}$ v.g./ml, 0.2 μl bilateral into PrL); AAV2/9-CMV-DIO-scrambled shRNA-eGFP (titer: 2.68 × $10^{12}$ v.g./ml, 0.2 μl bilateral into PrL). The sequence of *Tnfr1* shRNA: 5'-CCT CGT GCT TTC CAA GAT GAA-3'; Scrambled *shRNA*: 5'-GTT CTC CGA ACG TGT CAC GTA-3'. All viral vectors were aliquoted and stored at –80 °C until use.

For cannula implantation, the guide cannula (ID: 0.38 mm; RWD Life Science) together with a dummy cannula was unilaterally implanted inside the right PrL with the same coordination site as virus injection and cemented onto the skull with dental cement. Mice were then returned to their home cages and allowed to recover for at least 7 days before experiments. On the injection day, TNF-α was freshly prepared in $O_2$-saturated artificial cerebrospinal fluid (ACSF) at 5 ng/μl. TNF-α (1 μl), EN (1 μl, 50 μg/μl), or ACSF with the same volume was infused through an internal cannula (ID: 0.2 mm, RWD Life Science) controlled with the same microinjector at a rate of 0.1 μl/min. The capillary was slowly retracted 10 min after the injection.

For fiberoptic cannula implantation, the ceramic ferrule (diameter: 2.5 mm) with optic fiber (core diameter: 200 μm; NA: 0.37; length: 5 mm for the BLA and 3.5 mm for the l/vlPAG; Inper, China) was implanted bilaterally into the BLA (AP, -1.20 mm; DV, -4.55 mm; ML, ±3.20 mm) or unilaterally into the right l/vlPAG (AP, -4.85 mm; DV, -2.80 mm; ML, -0.35 mm).

**Verification of injection site and immunostaining**

Anesthetized mice were transcardially perfused with 0.9% saline followed by 4% paraformaldehyde (PFA) in 0.1 M phosphate-buffered saline (PBS). The brain was collected and fixed in a 4% PFA solution for at least 6 hr prior to transferring into 30% sucrose for dehydration. Brain blocks were then frozen in OCT at -80 °C freezer and sectioned into 30-μm-thick slices using a cryostat (CM1950, Leica). For injection site verification, slices were mounted with Vectashield

mounting medium (Vector lab), and images were captured using a confocal microscope (A1R, Nikon). For immunostaining, brain slices were permeabilized and blocked in 0.3% Triton X-100 and 5% BSA for 30 min, and incubated with primary antibodies at 4 °C overnight. After washing with PBS three times (10 min each), slices were incubated with Alexa Fluor-conjugated secondary antibodies (1:800, Jackson ImmunoResearch) for 1 h at room temperature, washed again, and mounted with Vectashield mounting medium (Vector lab). Images were captured using a confocal microscope (A1R, Nikon).

**Microprism implant assembly and implantation**

The microprism assemblies were constructed under a binocular microscope, in which a right-angle microprism (BK7 glass, 1.5 mm side length, Tower Optical) with an aluminum coating on hypotenuse was bonded to the center of a circular coverslip (Diameter: 3.5 mm; Thickness: 0.15 mm) using a UV-curing optical adhesive (Norland #81). Components were positioned in place with the help of vacuum clamping.

The microprism implantation was performed as described previously (6, 7). Briefly, anesthetized mice were head-fixed in a stereotaxic device (RWD Life Science. Inc, China). a ~4 mm circular craniotomy (coordinates of the center from bregma: AP, 1.8 mm; ML, 0.75 mm) was performed and the dura over the contralateral hemisphere of virus-injection site was gently removed with fine forceps. Great care was taken to minimize bleeding as diploic and emissary veins are prominent in the frontal region. Whenever possible, saline-soaked

surgical gelatin sponge was used to stop bleeding. The microprism assembly was positioned using vacuum, applied by a nozzle held in the stereotaxic frame. The front face and lower edge of the microprism were aligned parallel to the longitudinal fissure and the dorsal surface of the brain, respectively. The microprism was slowly lowered into the subdural space and did not stop until the coverslip slightly compressed the dorsal cortical surface, to minimize brain motion during the test. Veterinary adhesive (Vetbond, Fisher Scientific) was used to seal the gap between the implant and the skull. After retracting vacuum clamping, a custom-made circular titanium headplate was attached to the skull using dental cement (C&B Metabond, Parkell). The scalp incision was sutured and covered with erythromycin ointment.

**In-vivo two-photon $Ca^{2+}$ imaging and analysis**

Mice were placed under the objective by screwing the titanium head plate into a custom-built fork fixed to a solid metal base. All mice were trained to habituate this process for at least 1 week before the real experiment. Vascular landmarks and contours of the prism were first identified to relocate recording sites. During $Ca^{2+}$ imaging, the laser was tuned to 920 nm, with the power restricted below 20 mW. $Ca^{2+}$ images were acquired using a two-photon laser-scanning microscope (A1R, Nikon) with a water-immersed objective (40X, 0.8 NA, 3.5 mm working distance). Time-lapse $Ca^{2+}$ images with a resolution of 512 x 512 pixels were recorded in the PrL at 1 Hz for 5 min. If the substantial movement of mice was observed during image acquisition, the second set of images was obtained. In

the experiments detecting $Ca^{2+}$ activities in response to anxiogenic stimuli, a 15 ml-EP tube containing a cotton ball soaked with fox urine (Trap Shack company, USA) was rapidly placed ~ 1 cm away from the mice nostril for 1 s, or a foot shock (0.5 mA, 1s) generated by a customized electric shock module was given to the hindpaw of mice. Each type of stimuli was randomly given 10 times, with an interval of 1 min.

For analysis of neuronal $Ca^{2+}$ activity, the motion correction was first performed with a toolbox of EZ calcium-2.1.2 in MATLAB, followed by quantification of somatic calcium dynamics by ImageJ software (NIH) according to previous studies (8, 9). The fluorescent value ($F$) was obtained by averaging the intensities of pixels within the region of interest at each time point. Fluorescence dynamics $\Delta F/F_0$ was calculated as $(F - F_0)/F_0$, where the $F_0$ was the average of 10% minimum $F$ value (baseline fluorescence) over 5 min. Background fluorescence was subtracted from all the $F$ values. The integrated $Ca^{2+}$ activity was calculated by summation of all $\Delta F/F_0$, which are above three times the standard deviation (SD) of baseline fluorescence. A spontaneous $Ca^{2+}$ transient was identified as $(\Delta F/F_0)_{n-1} < (\Delta F/F_0)_n > (\Delta F/F_0)_{n+1}$ ($\Delta F/F_0 \geq 3 \times$ SD). In the experiment determining the responsive neurons to anxiogenic stimuli, the fluorescence signals were extracted 5s~3s before and 1s~5s after stimulation starts and analyzed by one-tailed Mann-Whitney U test. The neurons were defined responsive when $p < 0.05$.

**In-vivo optogenetic manipulation**

For optogenetic manipulation, mice were accommodated with the connection of implanted optic fiber to a laser generator (SLOC lasers, China) by a mating sleeve for one week before formal experiments. On the testing day, the 473-nm light (5ms, 20 Hz, 2-5 mW) or 594-nm light (constant, 5-8 mW) was delivered for 3 min during the “Light” phase only, controlled by an oscilloscope (Tektronix). The identical stimulus protocol was used for control mice.

**Electrophysiological recording**

The electrophysiological recording was performed as described previously (10). Briefly, mice were anesthetized with isoflurane, and brains were quickly collected and chilled in ice-cold modified artificial cerebrospinal fluid (ACSF) containing (in mM): 120 Choline-Cl, 2.5 KCl, 7 $MgCl_2$, 0.5 $CaCl_2$-2 $H_2O$, 1.25 $NaH_2PO_4$-1 $H_2O$, 25 $NaHCO_3$, and 10 D-glucose. Coronal brain slices (300 μm thickness) were sectioned in the same ice-cold modified ACSF using a VT-1000S vibratome (Leica, Germany) and kept in an incubation chamber that contains regular ACSF (in mM) (126 NaCl, 3 KCl, 1 $MgSO_4$-7 $H_2O$, 2 $CaCl_2$-2 $H_2O$, 1.25 $NaH_2PO_4$-1 $H_2O$, 26 $NaHCO_3$, and 10 D-glucose) at 32 °C for 30 min. Sections were then stayed at room temperature (24 ± 1 °C) for an additional 1 hr prior to recording. All solutions were saturated with 95% $O_2$ / 5% $CO_2$ (vol/vol).

For neuronal recording, slices were transferred to a recording chamber with continuous perfusion of regular ACSF at a rate of 2 ml/min. Neurons of interest were visualized with infrared optics using an upright microscope (BX51WIF, Olympus) equipped with an infrared-sensitive CCD camera (DAGE-

MTI, IR-1000E). Glass pipettes were pulled by a micropipette puller (P-97, Sutter instrument) with a resistance of 3-5 MΩ. Whole-cell recordings were made with MultiClamp 700B amplifier and 1440A digitizer (Molecular Devices).

For optogenetic light-induced action potential recording, neurons were recorded under current-clamp mode, with the pipette solution containing (in mM): 125 K-gluconate, 5 KCl, 10 HEPES, 0.2 EGTA, 1 $MgCl_2$, 4 Mg-ATP, 0.3 Na-GTP and 10 phosphocreatine (pH 7.40, 285 mOsm). The 473 nm-lights (5 ms/pulse, 1 s, 1-3 mW) at various frequencies (5-60 Hz) as indicated or 594 nm-lights (20 s) were given right above the neurons. Note that a positive current was injected into the neurons to elicit continuous firings during 594-nm lights inhibition experiment.

For optogenetic light-induced EPSC recording, neurons were held at -70 mV in the presence of 20 μM RS-95531, with the pipette solution containing (in mM): 125 Cs-methanesulfonate, 5 CsCl, 10 HEPES, 0.2 EGTA, 1 $MgCl_2$, 4 Mg-ATP, 0.3 Na-GTP, 10 phosphocreatine and 5 QX314 (pH 7.40, 285 mOsm). The 473 nm-lights (5 ms, 1-3 mV) were given at a frequency of 0.05 Hz to elicit EPSCs. TTX (1 μM) was added to the perfusion solution, followed by the addition of 4-aminopyridine (4-AP, 1 mM).

sEPSCs were recorded under the same condition as optogenetic light-induced EPSC recording. In the optogenetic experiment, the 594 nm-lights were continuously given for 1 min during the “Light” phase.

To record sIPSC, neurons were held at -70 mV in the presence of 20 μM CNQX and 100 μM AP-5, with the pipette solution containing (in mM): 140 CsCl,

10 Hepes, 0.2 EGTA, 1 $MgCl_2$, 4 Mg-ATP, 0.3 Na-GTP, 10 phosphocreatine and 5 QX314 (pH 7.40, 285 mOsm).

The excitability of PrL neurons was detected under current-clamp mode. A series of depolarizing pulses (from 0 pA to 120 pA, at a step of 20 pA) were injected to induce action potentials. The RMP and input resistance were detected by injecting a series of negative current pulses (0 pA, -50 pA, -100 pA). In some experiments, 20 μM CNQX, 50 μM AP-5, and 10 μM RS-95531 were added into the perfusion solution to block synaptic activity to record the intrinsic neuronal excitability.

To examine AMPAR and NMDAR mediated currents, EPSCs were recorded at -70 mV and +40 mV holding potentials in the presence of 20 μM RS-95531. A concentric bipolar electrode (CBARC75, FHC), ~ 200 μm away from the recording pipette, was used to elicit stimulations. AMPAR and NMDAR mediated currents were calculated as the peak amplitudes and 50 ms after the peak amplitude, respectively. For PPRs analysis, EPSCs were evoked at a holding potential of -70 mV in the presence of 20 μM RS-95531. Interval of paired stimulations was set at 50, 100, 200 and 300 ms. The value of ratios was defined as [p2 / p1] × 100, where p1 and p2 are the amplitude of the EPSCs evoked by the first and second pulse, respectively.

A peak-scaled non-stationary fluctuation analysis was performed on sEPSCs of $PrL^{BLA}$ neurons based on a previous report (11). Briefly, sEPSCs were selected following the criteria: stable baseline, fast rise time alignment, and the absence of spurious fluctuations during the sEPSC decay. Theoretically, the

decay phase of each EPSC varies from the mean decay of all the EPSCs when scaled to the same peak amplitude, and the plot of the variance of current amplitude during decay phase against the current amplitude is parabolic, of which the initial slope indicates the mean single-channel conductance. Accordingly, the variance of sEPSCs amplitudes during decay phase was plotted against the amplitudes during decay phase after the sEPSCs were peak scaled. The equation $\sigma^2 = iI - I^2/N + \sigma_b^2$ was used to fit the parabola. Note that *i* is the mean single-channel mediated current, *I* is the mean current, *N* is the number of channels activated at the peak, and $\sigma_b^2$ is the baseline variance. "*i*" was calculated as the slope of the linear fit of the first portion of the parabola. The single-channel conductance was estimated based on the reversal potential of eEPSCs (~0 mV) and the holding potential (-70 mV).

In all, series resistance was maintained below 20 MΩ and not compensated. Cells would be excluded if membrane potentials were positive more than -60 mV; or if series resistance fluctuated more than 20% of initial values. Data were filtered at 2 kHz and sampled at 10 kHz.

**Postsynaptic fractionation**

Postsynaptic fractions were prepared as described in previous reports (4, 12). In brief, brain tissues were immediately homogenized in 10 volumes of buffered sucrose (0.32 M sucrose and 4 mM HEPES/NaOH, containing protease inhibitors and phosphatase inhibitors, pH 7.4) with a glass-teflon homogenizer on ice. Homogenates were centrifuged at 1000 g for 10 min to remove nuclei and large

debris, supernatant (S1) collected and centrifuged at 10,000 g for 15 min to obtain the crude synaptosome containing pellet (P2). The P2 pellet was resuspended in dd$H_2O$ to dissociate the synaptosome within 10 s, and then balanced and incubated in a buffer on a rotator at 4 °C for 30 min. The resuspended P2 was centrifuged at 25,000 g for 20 min and the pellet containing synaptosomal membrane fraction (P3) resuspended in the discontinuous gradient sucrose solution (0.8,1.0,1.2 M, top to bottom), which was centrifuged at 150,000 g at 4 °C for 2 hr. Among the layered solution, a synaptosomal plasma membrane fraction resided at 1.0 M/1.2 M sucrose interface. Pipette out the fraction gently, then incubate with 1% Triton X-100 in 50 nM HEPES buffer at 4 °C for 30 min. After centrifugation at 25,000 g for 1hr, the supernatant was decanted, and the pellet containing postsynaptic membrane fractions was homogenized in RIPA buffer (Beyotime, P0013B) containing 1 mM PMSF (Beyotime, ST506). Samples were centrifuged at 14,000 g for 10 min, and the supernatant was collected. Protein concentrations were determined using a BCA protein assay kit (Pierce™, Catalog Number: 23225) with BSA (bovine serum albumin) as a standard. All samples were diluted to equal protein concentrations in sample loading buffer (6-8 μg/μl) and subjected to Western blotting.

**Western blotting**

As described previously with minor modification (13), the brains slices were cut using a VT-1000S vibratome (Leica, Germany). The PrL region was located under a fluorescence microscope (Eclipse Si, Nikon), and extracted using a fine

micro-forceps for homogenization. In some experiments, only the infection areas in the PrL region (green fluorescence), identified under the same fluorescence microscope, were collected for homogenization. Samples were subjected to SDS-PAGE and transferred to a PVDF membrane (Millipore, ISEQ00010). After blocking with 5% non-fat dry milk in TBS for 1 hr at room temperature, blots were probed overnight with primary antibody at 4 °C. After washing with TBST, membranes were incubated with horseradish peroxidase (HRP)-conjugated secondary antibodies (A0208 and A0216, Beyotime) for 1 hr at room temperature. Following washing, immunoreactive complex bands were visualized using enhanced chemiluminescence (Pierce) and captured using the Genesys imaging system (Gene Company Limited). Band densities of proteins of interest were normalized with a loading control.

**RNA sequencing**

RNA sequencing was performed as described previously (13). In brief, total RNA was extracted from PrL tissue using TRIzol reagent (Invitrogen, Carlsbad, USA) following the manufacturer's procedure. The amount and purity of RNA were quantified using NanoDrop ND-1000 (NanoDrop, USA). RNA integrity was assessed using Bioanalyzer 2100 (Agilent, USA) with RIN >7.0, followed by electrophoresis with denaturing agarose gel. Poly (A) RNA is purified from 1μg of RNA with Dynabeads Oligo (dT)25-61005 (Thermo Fisher, USA), fragmented into small pieces using Magnesium RNA Fragmentation Module (NEB, USA) under 94 °C for 5 – 7 min, and reverse-transcribed to generate the cDNA using

SuperScript™ II Reverse Transcriptase (Invitrogen, USA), which were then used to synthesize U-labeled second-stranded DNAs with E. coli DNA polymerase I (NEB, cat.m0209, USA), RNase H (NEB, USA) and dUTP Solution (Thermo Fisher, USA). An A-base is added to the blunt ends of each strand for ligation to the indexed adapters. Each adapter contains a T-base overhang for ligating the adapter to the A-tailed fragmented DNA. Following ligating the single- or dual-index adapters to the fragments, the size selection was performed with AMPureXP beads. After the treatment of the U-labeled second-stranded DNAs with the heat-labile UDG enzyme (NEB, USA), the ligated products are amplified with PCR with the following conditions: initial denaturation at 95 °C for 3 min; 8 cycles of denaturation at 98 °C for 15 s, annealing at 60 °C for 15 s, extension at 72 °C for 30 s, and final extension at 72 °C for 5 min. The average insert size for the final cDNA library was 300 ± 50 bp. The 2×150bp paired-end sequencing (PE150) on an Illumina Novaseq™ 6000 was performed following the vendor's recommended protocol.

**Transcriptome analysis**

For transcriptome analysis, fastp software (https://github.com/OpenGene/fastp) was used to exclude the reads containing adaptor contamination, base with low quality, and undetermined bases with default parameters and verify the quality of the sequence. The reads were mapped with HISAT2 (https://ccb.jhu.edu/software/hisat2) and assembled using StringTie (https://ccb.jhu.edu/software/stringtie) with default parameters. All transcriptomes

from all samples were then merged to reconstruct a comprehensive transcriptome with gffcompare (https://github.com/gpertea/gffcompare/). StringTie was used to perform expression level for mRNAs by calculating FPKM (FPKM = [total_exon_fragments / mapped_reads (millions) x exon_length (kB)]). The differentially expressed genes (DEGs) analysis was performed with a parametric F-test by comparing nested linear models (adjustive $p$ value < 0.05) with edgeR (R package) (https://bioconductor.org/packages/release/bioc/html/edgeR.html). GO and KEGG enrichment analysis of DEGs was performed with R pack clusterProfiler (https://bioconductor.org/packages/release/bioc/html/clusterProfiler.html). The raw dataset has been deposited in the Gene Expression Omnibus (GEO) database (GSE214204).

**Cytokine detection**

Mice were anesthetized with isoflurane and transcardially perfused with ice-cold 0.01M PBS to exclude the circulating blood. PrL tissues were then collected and transferred into a tube containing PBS for homogenization, followed by centrifugation at 4 °C with a velocity of 1,000 g for 10 min. Supernatants were collected and stored at −80 °C until use. The amounts of TNF-α, IL-1β, IL-10, and IL-6 were determined using Mouse QuantiCyto cytokines ELISA Kits (EMC102a, EMC001b, EMC005, EMC004, Neobioscience) following the manufacturer's instruction. Infinite M200 Pro NanoQuant was used to detect OD value. Data were analyzed by i-control 2.0 software.

**Single-cell RNA extraction**

Single-cell RNA extraction was performed according to a previous report with minor modification (14). In brief, the work surfaces were carefully cleaned with DNA-OFF (9036, Takara) and RNase Zap (AM9780, Life Technologies) to maintain an RNase-free environment. Autoclaved glass pipettes (O.D.: 1.5 mm, I.D.: 1.1 mm, Sutter Instruments) with a resistance of 1-2 MΩ were back-filled with ~1.0 μl of RNase-free solution containing (in mM): 125 K-gluconate, 12 KCl, 10 HEPES, 0.2 EGTA, 4 MgATP, 0.3 NaGTP, 10 Na phosphocreatine, 20 μg/ml glycogen, and 1 U/μl recombinant RNase inhibitor (2323A, Takara) (pH 7.40, 285 mOsm). Neurons of interest (visualized under a fluorescent microscope) were aspirated into the tip of pipettes by applying a light suction and then ejected using positive pressure into an RNase-free PCR tube with 4 μl of RNase-free lysis buffer supplied by the Single Cell Sequence-Specific Amplification Kit (P621-01, Vazyme), and then subjected to reverse transcription, amplification and further quantitative RT-PCR (qRT-PCR) following the manufacturer's instruction.

**qRT-PCR**

The qRT-PCR was performed as described previously (10). Briefly, fine pieces of PrL tissues were subjected to total RNA extraction using TRIzol reagent (15596-026, Invitrogen). RNA (1 μg) was reversely transcribed with oligo dT-primers using Maxima reverse transcriptase (EP0742, Fermentas) to generate cDNA, followed by qPCR with SYBR Green detection (K0222, Fermentas). Each sample

was assayed in triplicates, and each plate contained loading standards in duplicate. The mRNA levels of various genes were normalized to those of GAPDH. Primer sequences were: *Tnfa*: 5’- GGA ACA CGT CGT GGG ATA ATG -3’ and 5’- GGC AGA CTT TGG ATG CTT CTT -3’; *Il6*: 5’- TCC AGT TGC CTT CTT GGG AC -3’ and 5’- GTG TAA TTA AGC CTC CGA CTT G -3’; *Tnfr1*: 5’- GCA GTG TCT CAG TTG CAA GAC ATG TCG G -3’ and 5’- CGT TGG AAC TGG TTC TCC TTA CAG CCA C -3’; *Tnfr2*: 5’- ACA GTG CCC GCC CAG GTT GTC TTG -3’ and 5’- GCA GAA ATG TTT CAC ATA TTG GCC AGG AGG -3’; *GAPDH*, 5′- GGT TGT CTC CTG CGA CTT CA -3′ and 5′- CCA CCA CCC TGT TGC TGT AG -3′.

## References


1. Decosterd I, and Woolf CJ. Spared nerve injury: an animal model of persistent peripheral neuropathic pain. *Pain.* 2000;87(2):149-58.
2. Liu WZ, Zhang WH, Zheng ZH, Zou JX, Liu XX, Huang SH, et al. Identification of a prefrontal cortex-to-amygdala pathway for chronic stress-induced anxiety. *Nat Commun.* 2020;11(1):2221.
3. Bourquin AF, Süveges M, Pertin M, Gilliard N, Sardy S, Davison AC, et al. Assessment and analysis of mechanical allodynia-like behavior induced by spared nerve injury (SNI) in the mouse. *Pain.* 2006;122(1-2):14.e1-.
4. Sun XD, Chen WB, Sun D, Huang J, Li YQ, Pan JX, et al. Neogenin in Amygdala for Neuronal Activity and Information Processing. *J Neurosci.* 2018;38(44):9600-13.
5. Ren J, Lu CL, Huang J, Fan J, Guo F, Mo JW, et al. A Distinct Metabolically Defined Central Nucleus Circuit Bidirectionally Controls Anxiety-Related Behaviors. *J Neurosci.* 2022;42(11):2356-70.
6. Low RJ, Gu Y, and Tank DW. Cellular resolution optical access to brain regions in fissures: imaging medial prefrontal cortex and grid cells in entorhinal cortex. *Proc Natl Acad Sci U S A.* 2014;111(52):18739-44.
7. Moda-Sava RN, Murdock MH, Parekh PK, Fetcho RN, Huang BS, Huynh TN, et al. Sustained rescue of prefrontal circuit dysfunction by antidepressant-induced spine formation. *Science.* 2019;364(6436).

8. Zhou Y, Lai CSW, Bai Y, Li W, Zhao R, Yang G, et al. REM sleep promotes experience-dependent dendritic spine elimination in the mouse cortex. *Nat Commun.* 2020;11(1):4819.

9. Cantu DA, Wang B, Gongwer MW, He CX, Goel A, Suresh A, et al. EZcalcium: Open-Source Toolbox for Analysis of Calcium Imaging Data. *Front Neural Circuits.* 2020;14:25.

10. Sun XD, Li L, Liu F, Huang ZH, Bean JC, Jiao HF, et al. Lrp4 in astrocytes modulates glutamatergic transmission. *Nat Neurosci.* 2016;19(8):1010-8.

11. Valentinova K, Tchenio A, Trusel M, Clerke JA, Lalive AL, Tzanoulinou S, et al. Morphine withdrawal recruits lateral habenula cytokine signaling to reduce synaptic excitation and sociability. *Nat Neurosci.* 2019;22(7):1053-6.

12. Wang YN, Figueiredo D, Sun XD, Dong ZQ, Chen WB, Cui WP, et al. Controlling of glutamate release by neuregulin3 via inhibiting the assembly of the SNARE complex. *Proc Natl Acad Sci U S A.* 2018;115(10):2508-13.

13. Wang J, Huang J, Li YQ, Yao S, Wu CH, Wang Y, et al. Neuregulin 1/ErbB4 signaling contributes to the anti-epileptic effects of the ketogenic diet. *Cell Biosci.* 2021;11(1):29.

14. Chen YH, Hu NY, Wu DY, Bi LL, Luo ZY, Huang L, et al. PV network plasticity mediated by neuregulin1-ErbB4 signalling controls fear extinction. *Mol Psychiatry.* 2022;27(2):896-906.

## Supplemental Figures

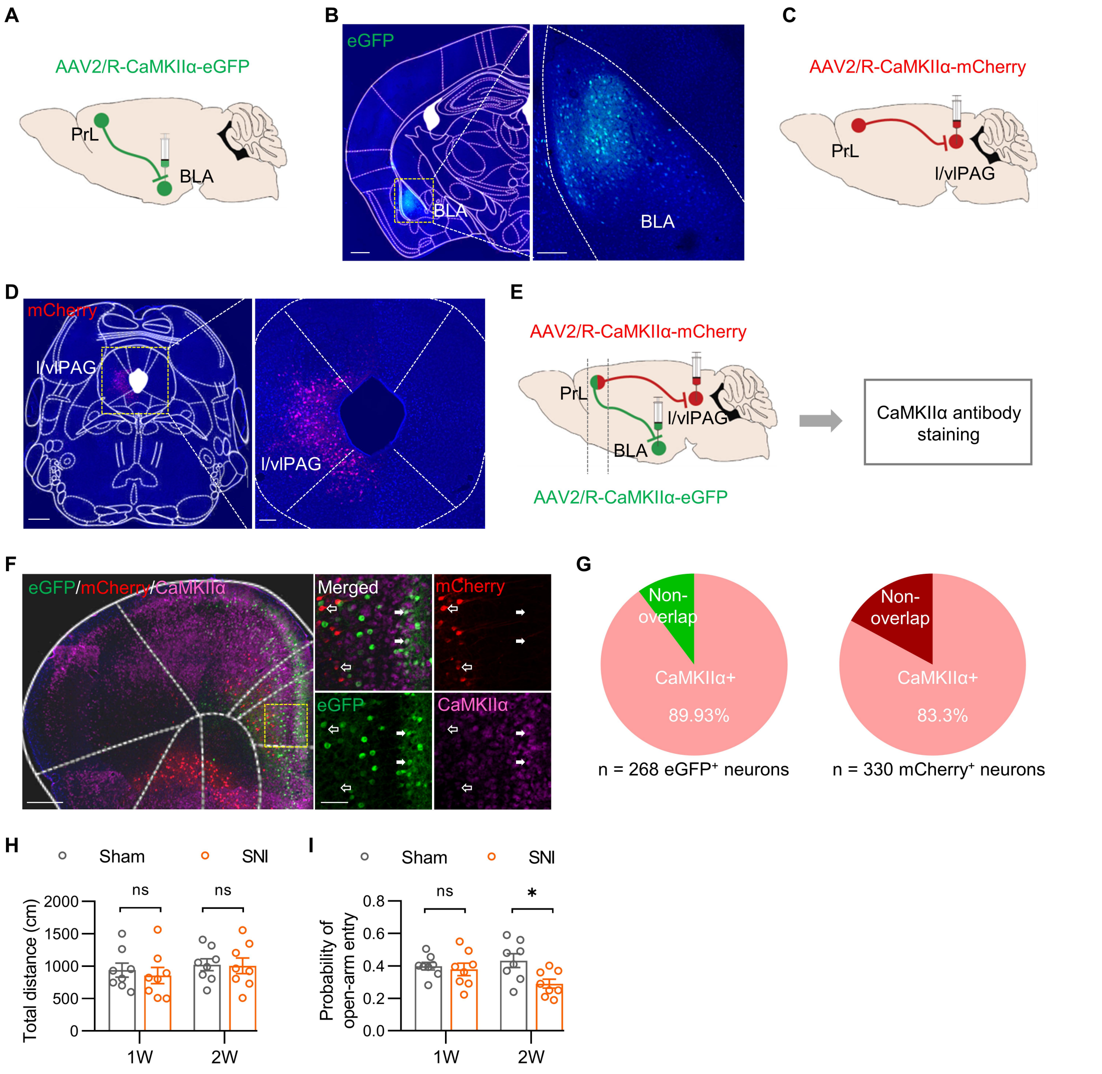


**Supplemental Figure 1 Characterization of virus-injection sites, the identity of PrL$^{BLA}$ and PrL$^{l/vlPAG}$ neurons, and chronic pain model.** (**A**) Schematic showing injection of retrograde AAV-CaMKIIα-eGFP virus into the BLA. (**B**) Representative image showing expression of eGFP in the BLA. Right, enlarged dotted area in the left image. Scale bars, 500 μm (Left) and 100 μm (Right). (**C**) Schematic showing injection of retrograde AAV-CaMKIIα-mcherry virus into the l/vlPAG. (**D**) Representative image showing expression of mcherry in the l/vlPAG.

Right, enlarged dotted area in the left image. Scale bars, 500 μm (Left) and 100 μm (Right). (**E**) Schematic showing injections of retrograde AAV-CaMKIIα-eGFP and AAV-CaMKIIα-mcherry virus into the BLA and l/vlPAG, respectively. Three weeks later, brain slices containing PrL were subjected to CaMKIIα antibody staining. (**F**) Representative image showing expression of eGFP, mcherry and CaMKIIα in the PrL. Right, enlarged dotted area in the left image. Hollow arrow, co-staining between mcherry and CaMKIIα; solid arrow, co-staining between eGFP and CaMKIIα.  Scale bars, 500 μm (Left) and 50 μm (Right). (**G**) Quantification of percentage of CaMKIIα in $eGFP^+$ or $mcherry^+$ neurons as in **F**. n = 268 $eGFP^+$ neurons from 3 mice; n = 330 $mcherry^+$ neurons from 3 mice. (**H**) Not changed total distance in SNI mice. n = 8 mice per group. (**I**) Reduced probability of open-arm entry two weeks after SNI surgery. n = 8 mice per group. Data were shown as mean ± SEM. **p* < 0.05; ns, no significant difference. Two-way repeated measures ANOVA followed by post-hoc Sidak's test (**H**, **I**).

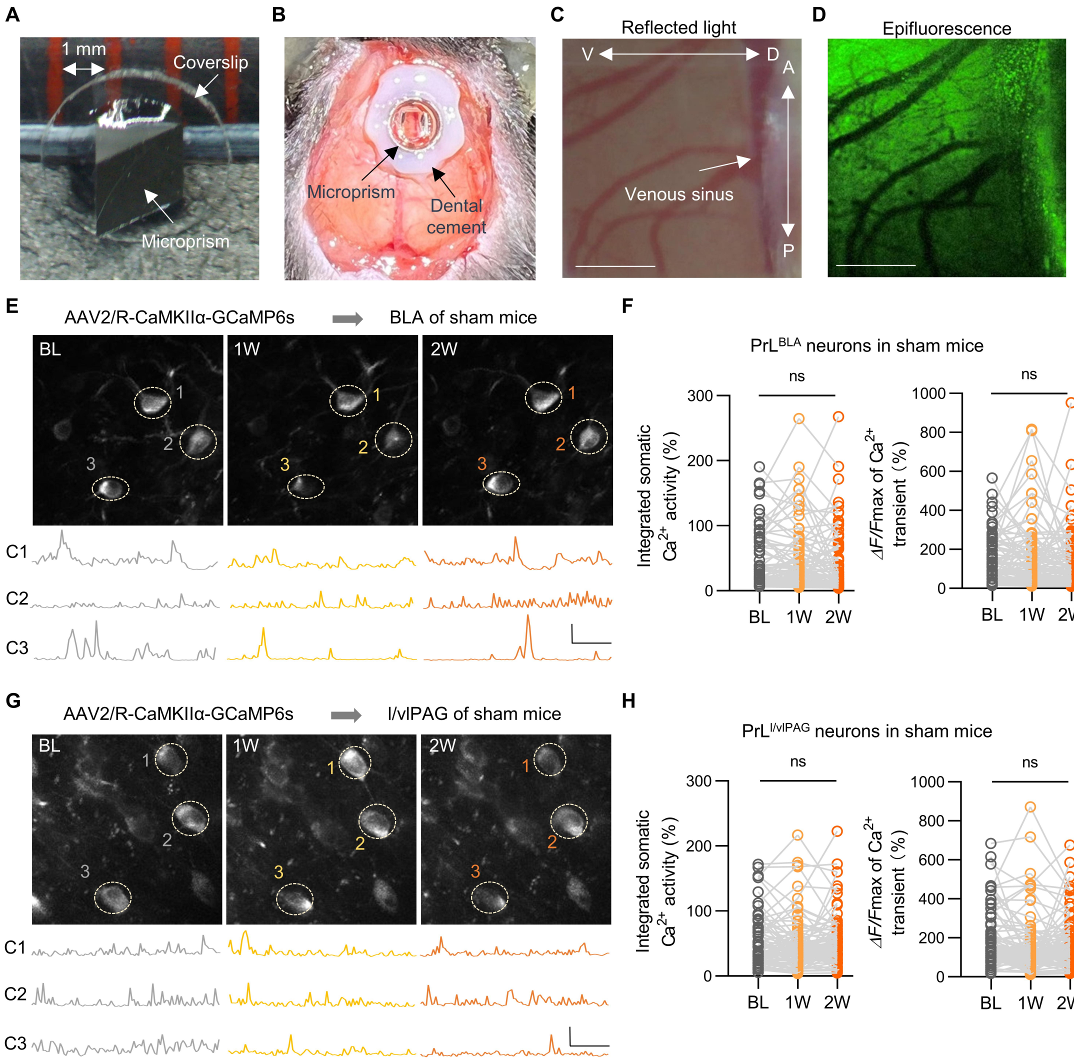


**Supplemental Figure 2 Unchanged Ca$^{2+}$ activity of PrL$^{BLA}$ and PrL$^{l/vlPAG}$ neurons in sham mice.** (**A**) Assembly of microprism implant. (**B**) implantation of microprism. (**C**) Widefield reflected light image of the mPFC through the implanted microprism. V, ventral; D, dorsal; A, anterior; P, posterior. Scale bar, 500 μm. (**D**) Epifluorescence image of the same region as in **C**. Scale bar, 500 μm. (**E**) Representative somatic Ca$^{2+}$ fluorescent images of PrL$^{BLA}$ neurons before (BL), one week (1W) and two weeks (2W) after sham surgery. Bottom, Ca$^{2+}$ fluorescent traces from the numbered neurons (C1, C2, C3) which are

circled in the upper images. Scale bars, 10 μm (upper); 500% *ΔF/F* and 20 s (bottom). (**F**) Not changed somatic $Ca^{2+}$ activity and peak amplitude of $Ca^{2+}$ transients of $PrL^{BLA}$ neurons in sham mice. n = 114 neurons from 5 sham mice. (**G**) Representative somatic $Ca^{2+}$ fluorescent images of $PrL^{l/vlPAG}$ neurons. Bottom, $Ca^{2+}$ fluorescent traces from the numbered neurons (C1, C2, C3) which are circled in the upper images. Scale bars, 10 μm (upper); 500% *ΔF/F* and 20 s (bottom). (**H**) Not changed somatic $Ca^{2+}$ activity and peak amplitude of $Ca^{2+}$ transients of $PrL^{l/vlPAG}$ neurons in sham mice. n = 94 neurons from 4 sham mice. Data were shown as aligned dot plots. ns, no significant difference. Friedman test (**F**, **H**).

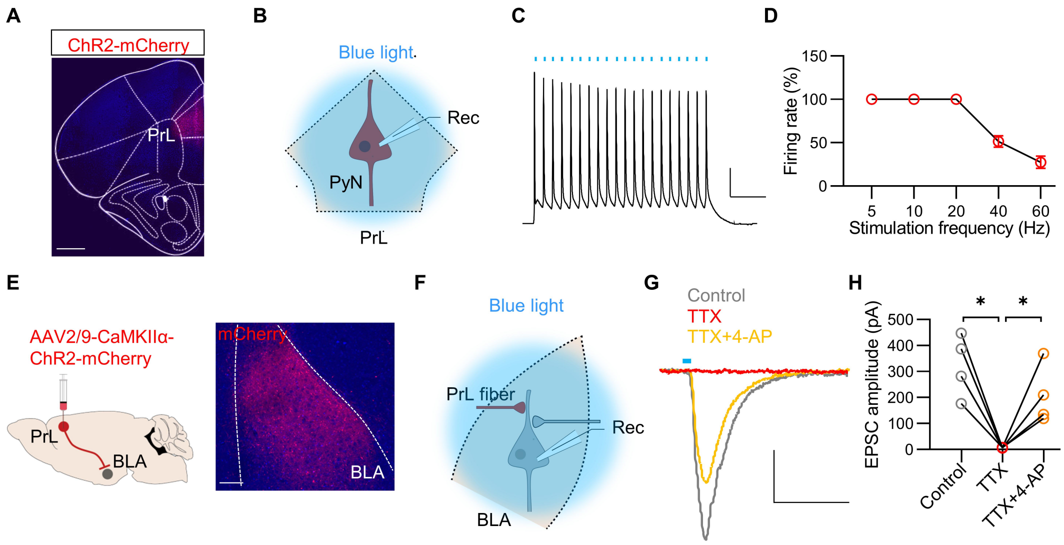


**Supplemental Figure 3 Characterizaiton of PrL-BLA circuit by optogenetics.** (**A**) Representative image showing expression of mcherry in the PrL. Scale bar, 500 μm. (**B**) Schematic showing recording of pyramidal neurons (PyN) in the PrL in response to blue light stimulation. Rec, record. (**C**) Representative trace showing induction of action potentials with a burst of blue lights at a frequency of 20 Hz. Scale bar, 20 mV and 200 ms. (**D**) Quantitative data of firing rates induced by blue lights at various frequencies. n = 9 neurons from 3 mice. (**E**) Schematic of viral injection (Left) and representative image showing expression of mcherry in the BLA (Right). Scale bar, 100 μm. (**F**) Schematic of neuronal recording in the BLA in response to optogenetic stimulation of PrL inputs. Rec, record. (**G**) Representative traces of EPSCs evoked by blue light without (Control) and with TTX or TTX+4-AP. Scale bars, 40 ms and 100 pA. (**H**) Quantitative data. n = 4 neurons from 3 mice. Data were shown as aligned dot plots. *$p < 0.05$. Repeated one-way ANOVA followed by post-hoc Turkey's test (**H**).

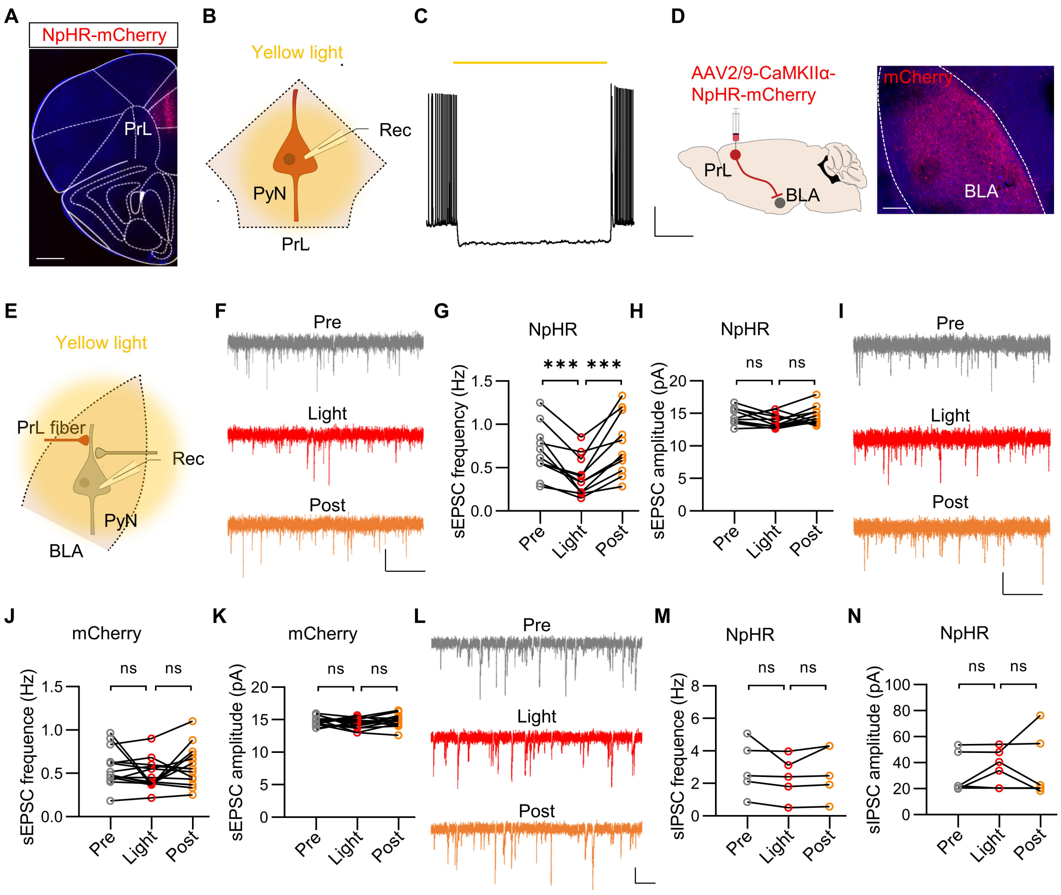


**Supplemental Figure 4 Characterization of the effects of optogenetic inhibition of PrL-BLA circuit on synaptic activity.** (**A**) Representative image showing expression of mcherry in the PrL. Scale bar, 500 μm. (**B**) Schematic showing recording of pyramidal neurons (PyN) in the PrL in response to yellow light inhibition. Rec, record. (**C**) Representative trace showing that a neuron was silenced by a period of yellow light. Scale bar, 5 s and 20 mV. (**D**) Schematic of viral injection (Left) and representative image showing expression of mcherry in the BLA (Right). Scale bar, 100 μm. (**E**) Schematic of recording in the BLA pyramidal neruons (PyN) in response to optogenetic inhibition of PrL inputs. Rec, record. (**F**) Representative sEPSC traces of BLA neurons with optogenetic inhibition of NpHR-expressing input. Scale bars, 2 s and 10 pA. (**G**) Decreased

sEPSC frequency. n = 12 neurons from 3 mice. (**H**) Unchanged sEPSC amplitude. n = 12 neurons from 3 mice. (**I**) Representative sEPSC traces of BLA neurons with optogenetic inhibition of mcherry-expressing input. Scale bars, 2 s and 10 pA. (**J**) Unchanged sEPSC frequency. n = 14 neurons from 3 mice. (**K**) Unchanged sEPSC amplitude. n = 14 neurons from 3 mice. (**L**) Representative sIPSC traces of BLA neurons with optogenetic inhibition of NpHR-expressing input. Scale bars, 500 ms and 10 pA. (**M**) Unchanged sIPSC frequency. n = 5 neurons from 3 mice. (**N**) Unchanged sIPSC amplitude. n = 5 neurons from 3 mice. Data were shown as aligned dot plots. ***$p$ < 0.001; ns, no significant difference. Repeated one-way ANOVA followed by post-hoc Turkey's test (**G**, **H**, **J**, **K**, **M**); Friedman test (**N**).

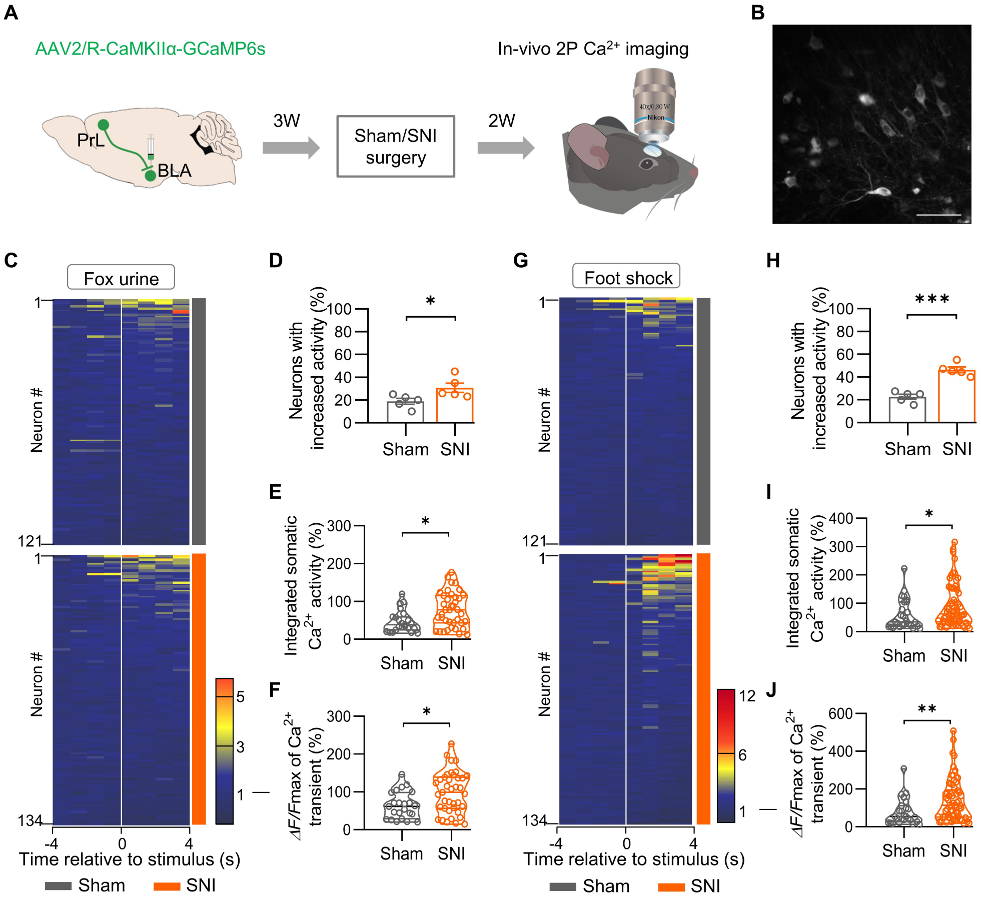


**Supplemental Figure 5 Characterization of the activities of PrL$^{BLA}$ neurons in response to fox urine and foot shock stimuli.** (**A**) Time scheme for in-vivo two photon $Ca^{2+}$ recording of PrL$^{BLA}$ neurons. 2P, two-photon. (**B**) Image of PrL$^{BLA}$ neurons expressing GCaMP6s. Scale bar, 30 μm. (**C**) Heatmaps showing the changes of somatic $Ca^{2+}$ activity of all neurons from sham (Top) and SNI (Bottom) mice in response to fox urine. Neurons were aligned from high to low $Ca^{2+}$ activity. (**D**) Increased proportions of “increased” PrL$^{BLA}$ neurons of PrL$^{BLA}$ neurons in response to fox urine. n = 5 mice per group. (**E**) Increased integrated somatic $Ca^{2+}$ activity of responsive PrL$^{BLA}$ neurons in SNI mice in response to fox urine. n = 23 and 39 neurons from 5 sham and 5 SNI mice, respectively. (**F**) Increased peak amplitude of $Ca^{2+}$ transients of responsive PrL$^{BLA}$ neurons in SNI

mice in response to fox urine. n = 23 and 39 neurons from 5 sham and 5 SNI mice, respectively. (**G**) Heatmaps showing the changes of somatic $Ca^{2+}$ activity of all neurons from sham (Top) and SNI (Bottom) mice in response to foot shock (0.5 mA, 1 s). (**H**) Proportions of $PrL^{BLA}$ neurons in response to foot shock. n = 5 mice per group. (**I**) Increased integrated somatic $Ca^{2+}$ activity of responsive $PrL^{BLA}$ neurons in SNI mice. n = 27 and 62 neurons from 5 sham and 5 SNI mice, respectively. (**J**) Increased peak amplitude of $Ca^{2+}$ transients of responsive $PrL^{BLA}$ neurons in SNI mice. n = 27 and 62 neurons from 5 sham and 5 SNI mice, respectively. Data were shown as mean ± SEM and aligned dot plots. *$p < 0.05$; **$p < 0.01$. student's *t* test (**D**, **E**, **F**, **H**); Mann-Whitney U test (**I**, **J**).

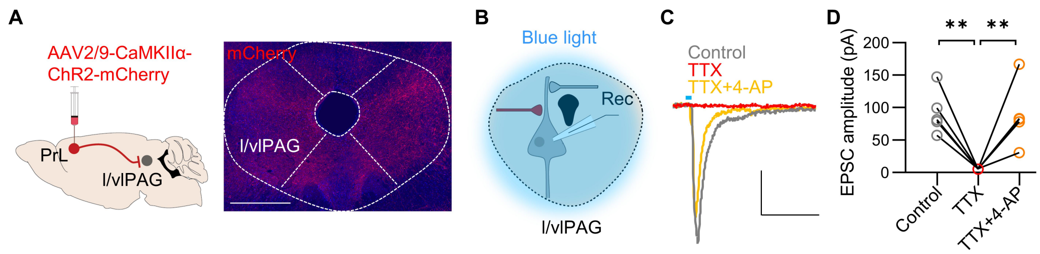


**Supplemental Figure 6 Characterizaiton of PrL-l/vlPAG circuit by optogenetics.** (**A**) Schematic of viral injection (Left) and representative image showing expression of mcherry in the l/vlPAG (Right). Scale bar, 500 μm. (**B**) Schematic of neuronal recording in the l/vlPAG in response to optogenetic stimulation of PrL inputs. Rec, record. (**C**) Representative traces of EPSCs evoked by blue light without (Control) and with TTX or TTX+4-AP. Scale bars, 40 ms and 30 pA. (**D**) Quantitative data. n = 5 neurons. Data were shown as aligned dot plot. $^{**}p < 0.01$. Repeated one-way ANOVA followed by post-hoc Turkey's test (**D**).

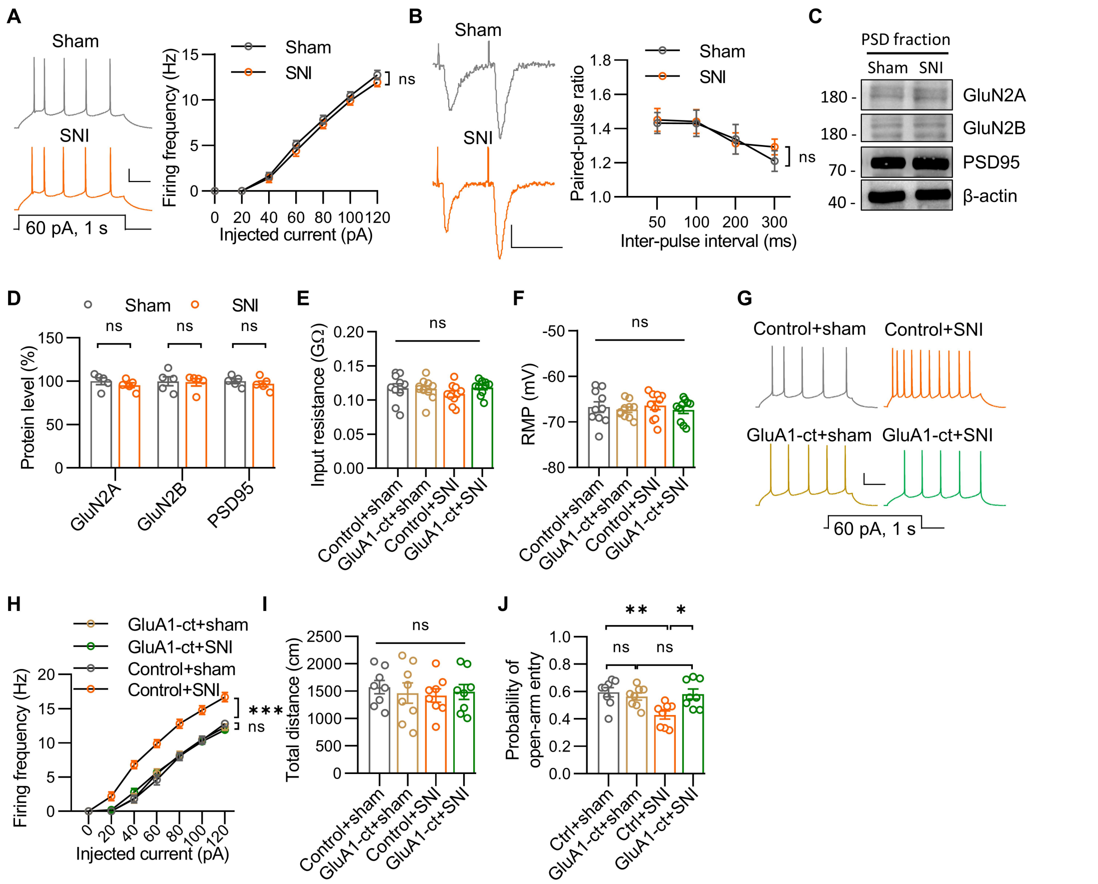


**Supplemental Figure 7 Characterization of electrophysiological properties of PrI$^{BLA}$ neurons and effects of GluA1-ct expression on neuronal excitability and behaviors.** (**A**) Unaltered intrinsic firing frequencies of PrL$^{BLA}$ neurons in SNI mice. Left, representative firing traces. Scale bars, 200 ms, 20 mV. Right, quantitative data. n = 11, 12 neurons from 3 sham, 3 SNI mice. (**B**) Comparable paired-pulse ratios of PrL$^{BLA}$ neurons. n = 20, 22 neurons from 4 sham, 4 SNI mice. (**C**) Representative western blots. (**D**) Quantitative data in **C**. n = 5 mice per group. (**E**, **F**) Comparable input resistance (**E**) and RMP (**F**) of PrL$^{BLA}$ neurons. n = 10 neurons from 3 mice per group. (**G**) Representative firing traces. Scale bars, 200 ms, 20 mV. (**H**) Reversed firing frequencies of PrL$^{BLA}$ neurons in SNI mice with expression of GluA1-ct. n = 10 neurons from 3 mice per

group. (**I**) Unchanged distance in OFT. n = 8 mice per group. (**J**) Reversed probability of open-arm entry in SNI mice by expressing GluA1-ct. n = 8 mice per group. Data were shown as mean ± SEM. *$p$ < 0.05; **$p$ < 0.01; ***$p$ < 0.001; ns, no significant difference. Two-way repeated-measures ANOVA (**A**, **B**, **H**); Student's *t* test (**D**); Mann-Whitney U test (**D**); One-way ANOVA followed by post-hot Turkey's test (**E**, **F**, **I**, **J**).

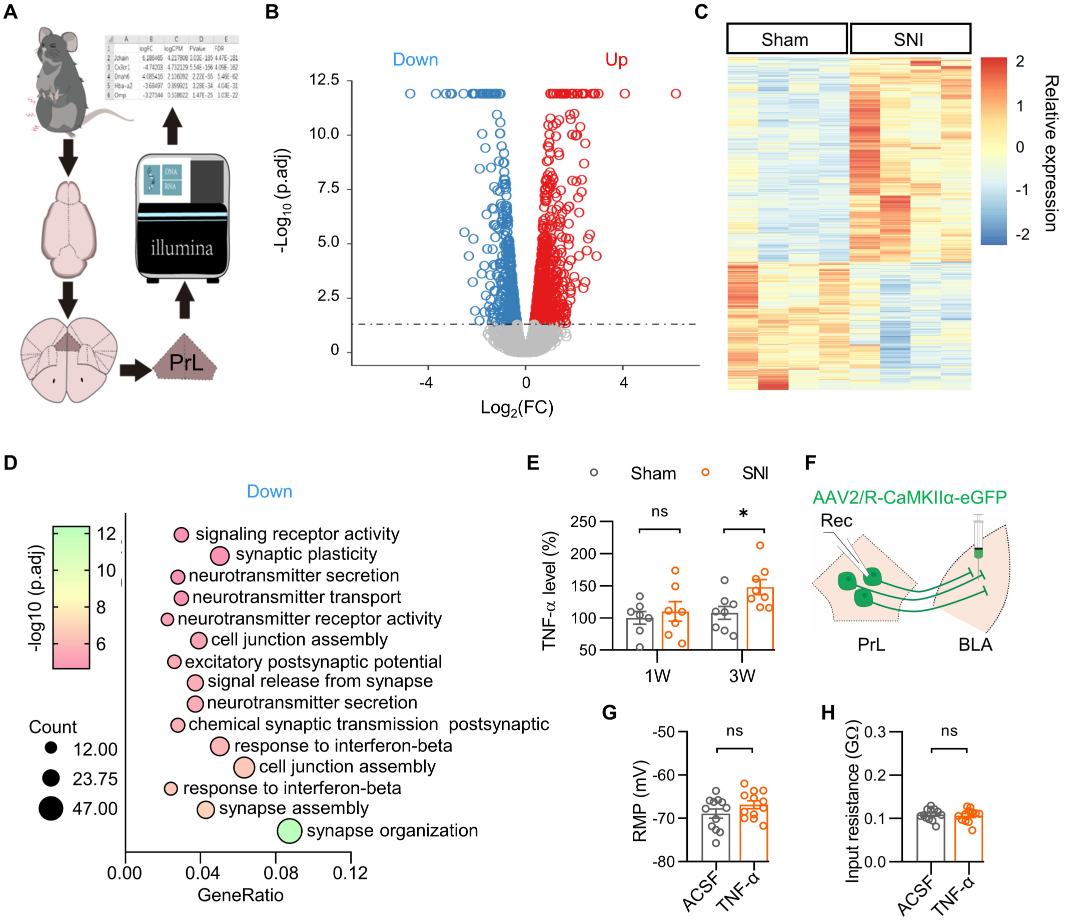


**Supplemental Figure 8 Analysis of RNAseq data obtained from PrL of chronic pain model and the effects of TNF-α on passive membrane properties of PrL$^{BLA}$ neurons.** (**A**) Schematic of experiments. PrL tissues of chronic pain model were collected and subjected to bulk RNA sequencing. (**B**) Volcano plots of differentially expressed genes (DEGs) between Sham and SNI mice. Blue and red dots indicate significantly down- and up-regulated genes, respectively. (**C**) Heatmap showing the transcriptome profiles of PrL in Sham and SNI mice. (**D**) Bubble plot of down-regulated genes-enriched biological process in GO analysis. (**E**) TNF-α levels in the PrL at one and three weeks after surgery. n = 7 mice per group for 1W, n = 8 mice per group for 3W. (**F**) Schematic of

electrophysiological recordings of $PrL^{BLA}$ neurons. Rec, record. (**G**) Unchanged RMP of $PrL^{BLA}$ neurons in mice with TNF-α injection into PrL. n = 12 neurons from 3 mice per group. (**H**) Unchanged input resistance of $PrL^{BLA}$ neurons in mice with TNF-α injection into PrL. n = 12 neurons from 3 mice per group. Data were shown as mean ± SEM. *$p < 0.05$; ns, no significant difference. Student's t test (**E**, **G**, **H**).

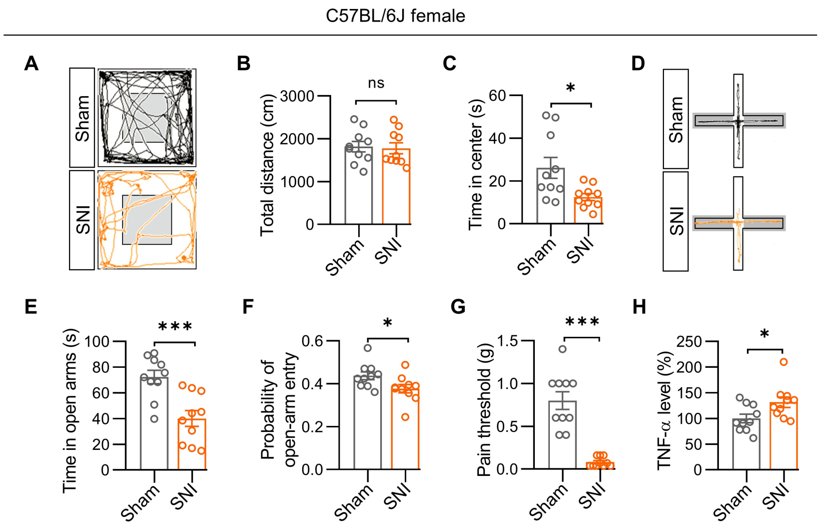


**Supplemental Figure 9 TNF-α level is increased in the PrL of female SNI mice.** (**A**) Representative traces of female mice travel in OFT. (**B**) Unchanged total distance that female mice traveled in OFT. n = 10 mice per group. (**C**) Reduced time in the center of female mice two weeks after SNI surgery. n = 10 mice per group. (**D**) Representative traces of female mice travel in EPM. (**E**) Reduced time in the open arms two weeks after SNI surgery. n = 10 mice per group. (**F**) Reduced probability of open-arms entry two weeks after SNI surgery. n = 10 mice per group. (**G**) Decreased mechanical pain threshold in female SNI mice at two weeks after surgery. n = 10 mice per group. (**H**) Increased TNF-α level in the PrL of SNI female mice at two weeks after surgery. n = 10 mice per group. Data were shown as mean ± SEM. *$p$ < 0.05; ***$p$ < 0.001; ns, no significant difference. Student's $t$ test (**B**, **C**, **E**, **F**, **G**, **H**).

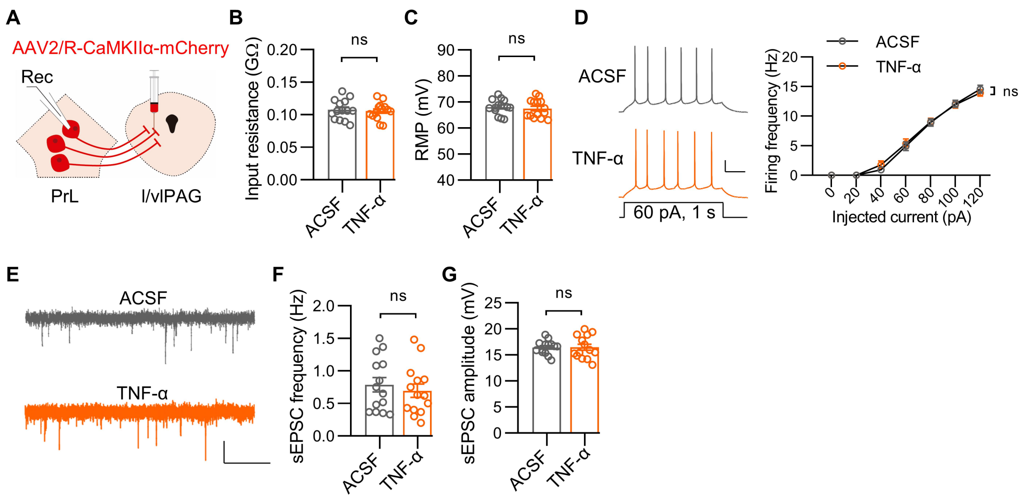


**Supplemental Figure 10 Characterization of the effect of TNF-α on PrL$^{\text{l/vlPAG}}$ neurons.** (**A**) Schematic showing viral injection into the l/vlPAG and recording of PrL$^{\text{l/vlPAG}}$ neurons. Rec, record. (**B**) Unchanged input resistance of PrL$^{\text{l/vlPAG}}$ neurons in mice with TNF-α injection into PrL. n = 14 neurons from 3 mice per group. (**C**) Unchanged RMP of PrL$^{\text{l/vlPAG}}$ neurons in mice with TNF-α injection into PrL. n = 12 neurons from 3 mice per group. (**D**) Comparable firing frequencies of PrL$^{\text{l/vlPAG}}$ neurons in response to current injections. Left, representative firing traces. Scale bars, 200 ms and 20 mV. Right, quantitative data. n = 14 neurons from 3 mice per group. (**E**) Representative sEPSC traces. Scale bars, 2 s and 10 pA. (**F**) Comparable sEPSC frequency of PrL$^{\text{l/vlPAG}}$ neurons in mice with TNF-α injection into PrL. n = 14 neurons from 3 mice per group. (**G**) Comparable sEPSC amplitude of PrL$^{\text{l/vlPAG}}$ neurons in mice with TNF-α injection into PrL. n = 14 neurons from 3 mice per group. Data were shown as mean ± SEM. ns, no significant difference. Student's *t* test (**B**, **C**, **G**); Two-way repeated-measures ANOVA (**D**); Mann-Whitney U test (**F**).

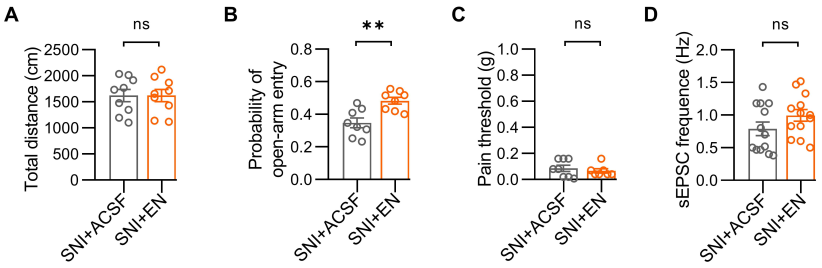


**Supplemental Figure 11 Characterization of the effects of EN in chronic pain.** (**A**) Unaltered total distance in OFT in mice with EN infusion into PrL. n = 9 mice per group. (**B**) Increased probability of open-arm entry in EPM in mice with EN infusion into PrL. n = 8 mice per group. (**C**) Not changed pain threshold by EN infusion in SNI mice. n = 8 mice for ACSF group, n = 7 mice for EN group. (**D**) Unaltered sEPSC frequency of $PrL^{BLA}$ neurons in SNI mice with EN infusion into PrL. n = 13 neurons from 4 mice per group. Data were shown as mean ± SEM. $^{**}p < 0.01$; ns, no significant difference. Student's *t* test (**A**, **B**, **D**); Mann-Whitney U test (**C**).

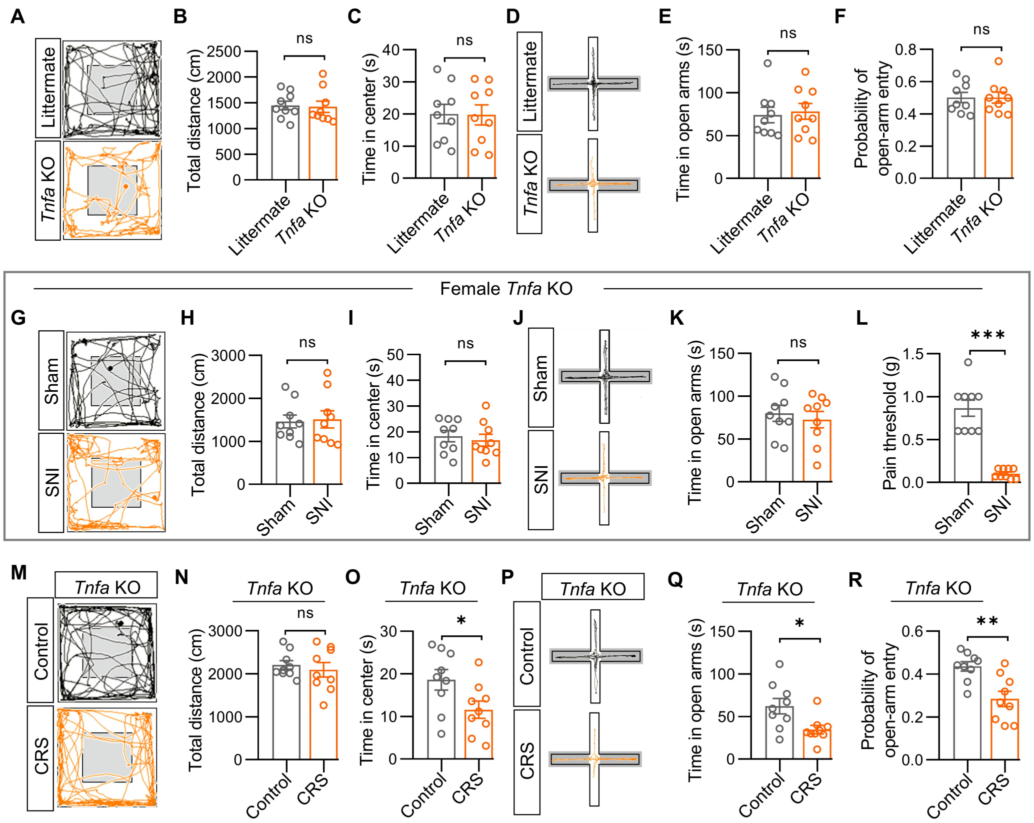


**Supplemental Figure 12 Characterization of the effects of *Tnfa* deletion on anxiety-like behaviors.** (**A**, **D**) Representative traces of mice travel in OFT (**A**) and in EPM (**D**). (**B**, **C**) Unchanged distance (**B**) and time in center (**C**) in OFT between *Tnfa* KO mice and their litermates. n = 9 mice per group. (**E**, **F**) Similar time in open arms (**E**) and probability of open-arm entry (**F**) between *Tnfa* KO mice and their litermates. n = 9 mice per group. (**G**, **J**) Representative traces of female *Tnfa* KO mice travel in OFT (**G**) and in EPM (**J**). (**H**, **I**) Unchanged distance (**H**) and time in center (**I**) in female *Tnfa* KO mice. n = 9 mice per group. (**K**) Unchanged time in the open arms in female *Tnfa* KO mice. n = 9 mice per group. (**L**) Decreased mechanical pain threshold in female *Tnfa* KO mice. n = 9 mice per group. (**M**, **P**) Representative travel traces of *Tnfa* KO mice in OFT (**M**) and in EPM (**P**) after CRS training. (**N**) Unchanged distance between CRS-

treated *Tnfa* KO mice and control *Tnfa* KO mice in OFT. n = 9 mice per group. (**O**) Decreased time in center between CRS-treated *Tnfa* KO mice and control *Tnfa* KO mice in OFT. n = 9 mice per group. (**Q**) Decreased time in open arms between CRS-treated *Tnfa* KO mice and control *Tnfa* KO mice in EPM. n = 9 mice per group. (**R**) Decreased probability of open-arm entry between CRS-treated *Tnfa* KO mice and control *Tnfa* KO mice in EPM. n = 9 mice per group. Data were shown as mean ± SEM. *$p < 0.05$; **$p < 0.01$; ***$p < 0.001$; ns, no significant difference. Student's *t* test (**B**, **C**, **E**, **F**, **H**, **I**, **K**, **N**, **O**, **Q**, **R**); Mann-Whitney U test (**L**).

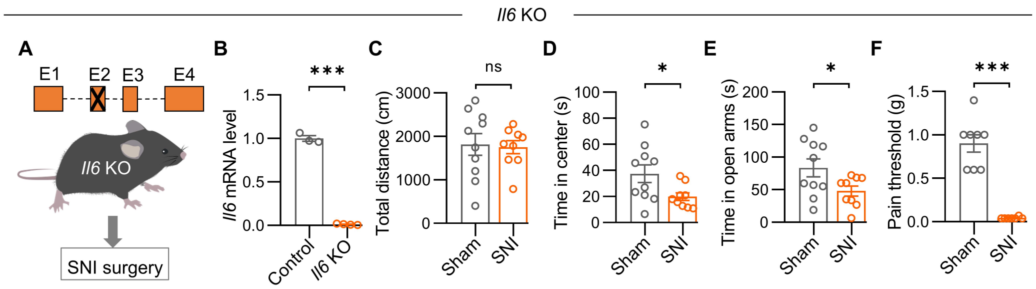


**Supplemental Figure 13 Characterization of the effects of *Il6* deletion in chronic pain.** (**A**) Schematic of *Il6* null (KO) mice which were subjected to SNI surgery. E, exon. (**B**) Undetectable *Il6* mRNA level in the PrL of *Il6* KO mice. n = 3 control mice, n = 4 *Il6* KO mice. (**C**) Unchanged total distance in OFT between Sham and SNI *Il6* KO mice. n = 10 and 9 mice for Sham and SNI groups, respectively. (**D**) Decreased time in center in OFT in SNI *Il6* KO mice. n = 10 and 9 mice for Sham and SNI groups, respectively. (**E**) Decreased time in open arms in EPM in SNI *Il6* KO mice. n = 10 and 9 mice for Sham and SNI groups, respectively. (**F**) Little effect of *Il6* deletion on pain threshold in SNI mice. n = 10 and 9 mice for Sham and SNI groups, respectively. Data were shown as mean ± SEM. *$p < 0.05$; ***$p < 0.001$; ns, no significant difference. Student's *t* test (**B**, **C**, **D**, **E**); Mann-Whitney U test (**F**).

**Video 1 Ca$^{2+}$ dynamics of PrL$^{BLA}$ neurons in sham mice.**

**Video 2 Ca$^{2+}$ dynamics of PrL$^{l/vlPAG}$ neurons in sham mice.**

**Video 3 Ca$^{2+}$ dynamics of PrL$^{BLA}$ neurons in SNI mice.**

**Video 4 Ca$^{2+}$ dynamics of PrL$^{l/vlPAG}$ neurons in SNI mice.**

## Statistical results

### Figure 1

(**E**) Two-way repeated measures ANOVA followed by post-hoc Sidak's test, group effect, $F_{(1,18)} = 117.2$, $p < 0.0001$. For 1W, t = 9.882, DF = 36, $p < 0.0001$; for 2W, t = 9.36, DF = 36, $p < 0.0001$. Time effect, $F_{(1,18)} = 2.729$, $p = 0.1159$; interaction effect, $F_{(1,18)} = 0.3239$, $p = 0.5763$.

(**G**) Two-way repeated measures ANOVA followed by post-hoc Sidak's test, group effect, $F_{(1,14)} = 7.232$, $p = 0.0176$. For 1W, t = 0.338, DF = 28, $p = 0.9313$; for 2W, t = 3.245, DF = 28, $p = 0.0061$. Time effect, $F_{(1,14)} = 4.13$, $p = 0.0615$; interaction effect, $F_{(1,14)} = 3.798$, $p = 0.0716$.

(**I**) Two-way repeated measures ANOVA followed by post-hoc Sidak's test, group effect, $F_{(1,14)} = 4.286$, $p = 0.0574$. For 1W, t = 0.4734, DF = 28, $p = 0.8701$; for 2W, t = 2.859, DF = 28, $p = 0.0158$. Time effect, $F_{(1,14)} = 0.0879$, $p = 0.7712$; interaction effect, $F_{(1,14)} = 4.157$, $p = 0.0608$.

(**L**, **M**) Friedman test with post-hoc Dunn's test, for integrated somatic $Ca^{2+}$ activity, $p < 0.0001$. BL vs 1W, $p = 0.0567$; BL vs 2W, $p < 0.0001$; 1W vs 2W, $p = 0.0197$. For peak amplitude of $Ca^{2+}$ transients, $p = 0.0002$. BL vs 1W, $p = 0.4144$; BL vs 2W, $p = 0.0001$; 1W vs 2W, $p = 0.0284$.

(**O**, **P**) Friedman test with post-hoc Dunn's test, for integrated somatic $Ca^{2+}$ activity, $p < 0.0001$. BL vs 1W, $p = 0.0309$; BL vs 2W, $p < 0.0001$; 1W vs 2W, $p = 0.0948$. For peak amplitude of $Ca^{2+}$ transients, $p < 0.0001$. BL vs 1W, $p = 0.0457$; BL vs 2W, $p < 0.0001$; 1W vs 2W, $p = 0.0795$.

**Figure 2**

(**C**) Repeated one-way ANOVA, for Sham+mcherry, $F_{(2,12)}$ = 1.5, *p* = 0.2621; for Sham+ChR2, $F_{(2,12)}$ = 0.5912, *p* = 0.569; Friedman test, for SNI+mcherry, *p* = 0.8724; for SNI+ChR2, *p* = 0.1111.

(**D**) Repeated one-way ANOVA followed by post-hoc Turkey's test. For Sham+mcherry, $F_{(2,12)}$ = 0.7855, *p* = 0.478; for Sham+ChR2, $F_{(2,12)}$ = 0.0202, *p* = 0.9801; for SNI+mcherry, $F_{(2,16)}$ = 0.3403, *p* = 0.7166; for SNI+ChR2, $F_{(2,16)}$ = 8.519, *p* = 0.003. Pre vs Light, *p* = 0.0032; Post vs Light, *p* = 0.0197.

(**E**) Repeated one-way ANOVA followed by post-hoc Turkey's test. For Sham+mcherry, $F_{(2,12)}$ = 0.5253, *p* = 0.6044; for Sham+ChR2, $F_{(2,14)}$ = 0.0473, *p* = 0.9539; for SNI+mcherry, $F_{(2,16)}$ = 0.442, *p* = 0.6503; for SNI+ChR2, $F_{(2,16)}$ = 9.63, *p* = 0.0018. Light vs Pre, *p* = 0.0013; Post vs Light, *p* = 0.0465.

(**G**) Repeated one-way ANOVA, for Sham+mcherry, $F_{(2,10)}$ = 0.0336, *p* = 0.9671; for Sham+ChR2, $F_{(2,10)}$ = 0.2088, *p* = 0.815. Friedman test, for SNI+mcherry, *p* = 0.3786; for SNI+ChR2, *p* = 0.2222.

(**H**) Repeated one-way ANOVA followed by post-hoc Turkey's test. For Sham+mcherry, $F_{(2,12)}$ = 0.0555, *p* = 0.9462; for Sham+ChR2, $F_{(2,12)}$ = 0.1458, *p* = 0.8659; for SNI+mcherry, $F_{(2,12)}$ = 0.1952, *p* = 0.8253; for SNI+ChR2, $F_{(2,12)}$ = 8.959, *p* = 0.0042. Pre vs Light, *p* = 0.0095; Post vs Light, *p* = 0.0075.

(**I**) Repeated one-way ANOVA, for Sham+mcherry, $F_{(2,12)}$ = 0.8546, *p* = 0.4498; for Sham+ChR2, $F_{(2,12)}$ = 0.6905, *p* = 0.5202. Friedman test, for SNI+mcherry, *p*

= 0.6197; for SNI+ChR2, $p = 0.0012$. Light vs Pre, $p = 0.0485$; Post vs Light, $p = 0.004$.

**Figure 3**

(**C**) Friedman test followed by post-hoc Dunn's test, for Sham+mcherry, $p = 0.321$; for Sham+ChR2, $p = 0.0247$, Pre vs Light, $p = 0.1351$; Post vs Light, $p > 0.9999$; for SNI+mcherry, $p > 0.9999$; for Sham+ChR2, $p = 0.0005$, Pre vs Light, $p = 0.004$, Post vs Light, $p = 0.0485$.

(**D**) Friedman test, for Sham+mcherry, $p = 0.3046$; for SNI+mcherry, $p = 0.5023$; for Sham+ChR2, $p = 0.6197$. Repeated one-way ANOVA, for Sham+ChR2, $F_{(2,12)} = 0.1408$, $p = 0.7738$.

(**F**) Friedman test followed by post-hoc Dunn's test, for Sham+mcherry, $p = 0.2387$; for Sham+NpHR, $p = 0.0009$, Pre vs Light, $p = 0.0063$, Post vs Light, $p = 0.0334$; for SNI+mcherry, $p > 0.9999$; for SNI+ChR2, $p = 0.0055$, Pre vs Light, $p = 0.0693$, Post vs Light, $p = 0.0334$.

(**G**) Repeated one-way ANOVA. For Sham+mcherry, $F_{(2,12)} = 1.376$, $p = 0.2896$; for Sham+ChR2, $F_{(2,12)} = 0.5011$, $p = 0.618$; for SNI+mcherry, $F_{(2,12)} = 1.078$, $p = 0.3711$; for SNI+ChR2, $F_{(2,12)} = 0.0352$, $p = 0.9655$.

**Figure 4**

(**B**) Mann-Whitney U test, $p = 0.6209$.

(**C**) Student's t test, $t_{(27)} = 0.1021$, $p = 0.9194$.

(**D**) Two-way repeated-measures ANOVA, for group effect, $F_{(1,44)} = 10.68$, $p = 0.0021$; for current effect, $F_{(6,264)} = 437.2$, $p < 0.0001$; for interaction, $F_{(6,264)} = 3.102$, $p = 0.0059$.

(**F**) Student's *t* test, $t_{(28)} = 0.2996$, $p$ = 0. 7667.

(**G**) Mann-Whitney U test, $p = 0.0002$.

(**I**) Mann-Whitney U test, $p = 0.8578$.

(**J**) Student's *t* test, $t_{(33)} = 0.4512$, $p = 0.6548$.

**Figure 5**

(**B**) Student's *t* test, $t_{(22)} = 1.016$, $p = 0.3205$.

(**C**) Student's *t* test, $t_{(22)} = 0.2446$, $p = 0.8091$.

(**D**) Two-way repeated-measures ANOVA, for group effect, $F_{(1,22)} = 27.0$, $p < 0.0001$; for current effect, $F_{(6,132)} = 556.7$, $p < 0.0001$; for interaction, $F_{(6,132)} = 13.76$, $p < 0.0001$.

(**F**) Student's *t* test, $t_{(28)} = 0.127$, $p = 0.8999$.

(**G**) Student's *t* test, $t_{(28)} = 1.812$, $p = 0.0807$.

(**I**) Student's *t* test, $t_{(32)} = 5.623$, $p < 0.0001$.

(**J**) Student's *t* test, $t_{(32)} = 1.935$, $p = 0.0619$.

**Figure 6**

(**B**) For NMDAR-mediated currents, student's *t* test, $t_{(32)} = 0.23$, $p = 0.8196$; for AMPAR-mediated currents, Mann-Whitney U test, $p = 0.0007$.

(**D**) Student's *t* test, $t_{(17)} = 2.345$, $p = 0.0314$.

(**E**) Mann-Whitney U test, $p = 0.2775$.

(**G**) Student's *t* test, for GluA1, $t_{(8)} = 8.163$, $p < 0.001$; for GluA2, $t_{(8)} = 1.333$, $p = 0.2193$; for GluN1, $t_{(8)} = 0.9311$, $p = 0.379$.

**Figure 7**

(**E**) One-way ANOVA followed by post-hoc Turkey's test, $F_{(3,8)} = 22.07$, $p = 0.0003$. Control+sham vs GluA1-c+sham, $p = 0.8433$; Control+sham vs Control+SNI, $p = 0.0016$; Control+SNI vs GluA1-c+SNI, $p = 0.0005$; GluA1-c+sham vs GluA1-c+SNI, $p = 0.9857$.

(**H**) Kruskal-Wallis test, $p = 0.5452$.

(**I**) Kruskal-Wallis test followed by post-hoc Dunn's test, $p < 0.0001$. Control+sham vs GluA1-ct+sham, $p > 0.999$; Control+sham vs Control+SNI, $p = 0.0002$; Control+SNI vs GluA1-ct+SNI, $p = 0.0076$; GluA1-ct+sham vs GluA1-ct+SNI, $p > 0.999$.

(**K**) Kruskal-Wallis test followed by post-hoc Dunn's test, $p = 0.0015$. Control+sham vs GluA1-ct+sham, $p > 0.999$; Control+sham vs Control+SNI, $p = 0.0091$; Control+SNI vs GluA1-ct+SNI, $p = 0.0076$; GluA1-ct+sham vs GluA1-ct+SNI, $p > 0.999$.

(**M**) One-way ANOVA followed by post-hoc Turkey's test, $F_{(3,28)} = 6.557$, $p = 0.0017$. Control+sham vs GluA1-ct+sham, $p = 0.9681$; Control+sham vs Control+SNI, $p = 0.0095$; Control+SNI vs GluA1-ct+SNI, $p = 0.0087$; GluA1-ct+sham vs GluA1-ct+SNI, $p = 0.9752$.

**Figure 8**

(**C**) Student's *t* test, for TNF-α, $t_{(10)} = 3.345$, $p = 0.0074$; for IL-1β, $t_{(10)} = 0.168$, $p = 0.87$; for IL-10, $t_{(10)} = 1.191$, $p = 0.261$; for IL-6, $t_{(10)} = 0.3713$, $p = 0.7182$.

(**E**) Mann-Whitney U test, $p = 0.9782$.

(**G**) Student's *t* test, $t_{(14)} = 4.882$, $p = 0.0002$.

(**I**) Student's *t* test, $t_{(14)} = 5.656$, $p < 0.0001$.

(**J**) Two-way repeated measures ANOVA, for group effect, $F_{(1,22)} = 18.05$, $p = 0.0003$; for current effect, $F_{(6,132)} = 585.2$, $p < 0.0001$; for interaction, $F_{(6,132)} = 7.22$, $p < 0.0001$.

(**L**) Student's *t* test, $t_{(27)} = 3.181$, $p = 0.0037$.

(**M**) Student's *t* test, $t_{(27)} = 1.886$, $p = 0.0701$.

**Figure 9**

(**C**) Student's *t* test, $t_{(16)} = 2.903$, $p = 0.0104$.

(**E**) Student's *t* test, $t_{(14)} = 2.418$, $p = 0.0298$.

(**G**) Student's *t* test, $t_{(28)} = 5.256$, $p < 0.0001$.

(**H**) Two-way repeated-measures ANOVA, for group effect, $F_{(1,31)} = 19.75$, $p = 0.0001$; for current effect, $F_{(6,186)} = 421.5$, $p < 0.0001$; for interaction, $F_{(6,186)} = 15.19$, $p < 0.0001$.

(**I**) Student's t test, $t_{(6)} = 5.128$, $p = 0.0022$.

(**J**) Mann-Whitney U test, $p = 0.0006$.

(**L**) Student's *t* test, $t_{(13)} = 0.7935$, $p = 0.4417$.

(**N**) Student's *t* test, $t_{(13)} = 0.3678$, $p = 0.7189$.

(**P**) Student's *t* test, $t_{(24)} = 0.15$, $p = 0.882$.

(**Q**) Student's *t* test, $t_{(24)} = 1.386$, $p = 0.1784$.

(**R**) Two-way repeated-measures ANOVA, for group effect, $F_{(1,22)} = 2.56$, $p = 0.1239$; for current effect, $F_{(6,132)} = 687.1$, $p < 0.0001$; for interaction, $F_{(6,132)} = 0.9128$, $p = 0.4879$.

**Figure 10**

(**A**) Mann-Whitney U test, p < 0.0001.

(**D**) Student's *t* test, $t_{(6)} = 3.181$, $p = 0.0191$.

(**E**) Kruskal-Wallis test followed by Dunn's test, $p < 0.0001$. For Control+sham vs *Tnfr1*-KD+sham, $p > 0.999$; for *Tnfr1*-KD+sham vs *Tnfr1*-KD+SNI, $p = 0.0088$; for Control+sham vs Control+SNI, $p = 0.0002$; for Control+SNI vs *Tnfr1*-KD+SNI, $p > 0.999$.

(**G**) Kruskal-Wallis test followed by Dunn's test, $p = 0.0011$. For Control+sham vs *Tnfr1*-KD+sham, $p > 0.999$; for *Tnfr1*-KD+sham vs *Tnfr1*-KD+SNI, $p > 0.9999$; for Control+sham vs Control+SNI, $p = 0.0099$; for Control+SNI vs *Tnfr1*-KD+SNI, $p = 0.0035$.

(**I**) One-way ANOVA followed by post-hoc Turkey's test, $F_{(3,28)} = 5.731$, $p = 0.0035$. Control+sham vs *Tnfr1*-KD+sham, $p = 0.9961$; Control+sham vs Control+SNI, $p = 0.0054$; Control+SNI vs *Tnfr1*-KD+SNI, $p = 0.0377$; *Tnfr1*-KD+sham vs *Tnfr1*-KD+SNI, $p = 0.9372$.

(**K**) Kruskal-Wallis test followed by Dunn's test, $p < 0.0001$. Control+sham vs *Tnfr1*-KD+sham, $p > 0.999$; Control+sham vs Control+SNI, $p < 0.0001$;

Control+SNI vs *Tnfr1*-KD+SNI, $p = 0.0162$; *Tnfr1*-KD+sham vs *Tnfr1*-KD+SNI, $p > 0.999$.

(**L**) Two-way repeated-measures ANOVA, For Control+sham vs. Control+SNI, group effect, $F_{(1,28)} = 69.68$, $p < 0.0001$; current effect, $F_{(6,168)} = 635.8$, $p < 0.0001$; interaction, $F_{(6,168)} = 27.35$, $p < 0.0001$. For *Tnfr1*-KD+sham vs. *Tnfr1*-KD+SNI, group effect, $F_{(1,28)} = 0.939$, $p = 0.3408$; current effect, $F_{(6,168)} = 338.7$, $p < 0.0001$; interaction, $F_{(6,168)} = 1.01$, $p = 0.4208$.

**Supplemental Figure 1**

(**H**) Two-way repeated measures ANOVA, group effect, $F_{(1,14)} = 0.1215$, $p = 0.7326$; time effect, $F_{(1,14)} = 3.858$, $p = 0.0697$; interaction effect, $F_{(1,14)} = 0.307$, $p = 0.5883$.

(**I**) Two-way repeated measures ANOVA followed by post-hoc Sidak's test, group effect, $F_{(1,14)} = 5.708$, $p = 0.0315$. For 1W, $p = 0.8898$; for 2W, $p = 0.0119$. Time effect, $F_{(1,14)} = 0.6684$, $p = 0.4273$; interaction effect, $F_{(1,14)} = 3.298$, $p = 0.0908$.

**Supplemental Figure 2**

(**F**) Friedman test, for somatic $Ca^{2+}$ activity, $p = 0.0625$; for peak amplitude of $Ca^{2+}$ transients, $p = 0.1251$.

(**H**) Friedman test, for somatic $Ca^{2+}$ activity, $p = 0.2674$. For peak amplitude of $Ca^{2+}$ transients, $p = 0.1283$.

**Supplemental Figure 3**

(**H**) Repeated one-way ANOVA followed by post-hoc Turkey's test, $F_{(2,6)} = 20.06$, $p = 0.0022$; Control vs TTX, $p = 0.0019$; TTX vs TTX+4-AP, $p = 0.0169$.

**Supplemental Figure 4**

(**G**) Repeated one-way ANOVA followed by post-hoc Turkey's test, $F_{(2,22)} = 16.28$, $p < 0.0001$; Pre vs Light, $p = 0.0009$; Light vs Post, $p < 0.0001$.

(**H**) Repeated one-way ANOVA, $F_{(2,22)} = 1.47$, $p = 0.2516$.

(**J**) Repeated one-way ANOVA, $F_{(2,26)} = 1.728$, $p = 0.1974$.

(**K**) Repeated one-way ANOVA, $F_{(2,26)} = 1.416$, $p = 0.2608$.

(**M**) Repeated one-way ANOVA, $F_{(2,8)} = 2.357$, $p = 0.1568$.

(**N**) Friedman test, $p = 0.9537$.

**Supplemental Figure 5**

(**D**) Student's *t* test, $t_{(8)} = 2.485$, $p = 0.0378$.

(**E**) Student's *t* test, $t_{(60)} = 2.925$, $p = 0.0049$.

(**F**) Student's *t* test, $t_{(60)} = 2.733$, $p = 0.0082$.

(**H**) Student's *t* test, $t_{(8)} = 7.261$, $p < 0.0001$.

(**I**) Mann-Whitney U test, $p = 0.0232$.

(**J**) Mann-Whitney U test, $p = 0.0038$.

**Supplemental Figure 6**

(**D**) Repeated one-way ANOVA followed by post-hoc Turkey's test, $F_{(2,8)}$ = 18.17, $p$ = 0.0011; Control vs TTX, $p$ = 0.0017; TTX vs TTX+4-AP, $p$ = 0.0024.

**Supplemental Figure 7**

(**A**) Two-way repeated-measures ANOVA, for group effect, $F_{(1,21)}$ = 0.6853, $p$ = 0.4171; for current effect, $F_{(6,126)}$ = 722.3, $p$ < 0.0001; for interaction, $F_{(6,126)}$ = 0.7535, $p$ = 0.6078.

(**B**) Two-way repeated measures ANOVA, group effect, $F_{(1,40)}$ = 0.0893, $p$ = 0.7666; interval effect, $F_{(3,120)}$ = 6.986, $p$ = 0.0002; interaction effect, $F_{(3,120)}$ = 0.4172, $p$ = 0.741.

(**D**) For GluN2A, Student's *t* test, $t_{(8)}$ = 0.9311, $p$ = 0.379; for GluN2B, Mann-Whitney U test, $p$ = 0.8413; for PSD95, student's *t* test, $t_{(8)}$ = 0.7162, $p$ = 0.4943.

(**E**) One-way ANOVA, $F_{(3,36)}$ = 0.7398, $p$ = 0.5353.

(**F**) One-way ANOVA, $F_{(3,36)}$ = 0.2665, $p$ = 0.8491.

(**H**) Two-way repeated-measures ANOVA, For Control+sham vs. Control+SNI, group effect, $F_{(1,18)}$ = 34.25, $p$ < 0.0001; current effect, $F_{(6,108)}$ = 565.6, $p$ < 0.0001; interaction, $F_{(6,108)}$ = 15.98, $p$ < 0.0001. For GluA1-ct+sham vs. GluA1-ct+SNI, group effect, $F_{(1,18)}$ = 0.0296, $p$ = 0.8654; current effect, $F_{(6,108)}$ = 508.6, $p$ < 0.0001; interaction, $F_{(6,108)}$ = 0.9876, $p$ = 0.4374.

(**I**) One-way ANOVA, $F_{(3,28)}$ = 0.2001, $p$ = 0.8954.

(**J**) One-way ANOVA followed by post-hot Turkey's test, $F_{(3,28)}$ = 5.814, $p$ = 0.0032. Control+Sham vs GluA1-ct+Sham, $p$ = 0.8916; Control+Sham vs

Control+SNI, $p$ = 0.005; Control+SNI vs GluA1-ct+SNI, $p$ = 0.0102; GluA1-ct+Sham vs GluA1-ct+SNI, $p$ = 0.9737.

**Supplemental Figure 8**

(**E**) Student's t test, for 1w, $t_{(12)} = 0.5487$, $p = 0.5932$; for 3w, $t_{(14)} = 2.599$, $p = 0.021$.

(**G**) Student's t test, $t_{(22)} = 1.462$, $p = 0.1579$.

(**H**) Student's *t* test, $t_{(22)} = 0.3732$, $p = 0.7126$.

**Supplemental Figure 9**

(**B**) Student's *t* test, $t_{(18)} = 0.2206$, $p = 0.8279$.

(**C**) Student's *t* test, $t_{(18)} = 2.652$, $p = 0.0162$.

(**E**) Student's *t* test, $t_{(18)} = 3.985$, $p = 0.0009$.

(**F**) Student's *t* test, $t_{(18)} = 2.266$, $p = 0.036$.

(**G**) Student's *t* test, $t_{(18)} = 6.855$, $p < 0.0001$.

(**H**) Student's *t* test, $t_{(18)} = 2.394$, $p = 0.0278$.

**Supplemental Figure 10**

(**B**) Student's *t* test, $t_{(26)} = 0.4011$, $p = 0.6917$.

(**C**) Student's t test, $t_{(26)} = 0.4897$, $p = 0.6284$.

(**D**) Two-way repeated-measures ANOVA, for group effect, $F_{(1,26)} = 0.0264$, $p = 0.8723$; for current effect, $F_{(6,156)} = 513.4$, $p < 0.0001$; for interaction, $F_{(6,156)} = 0.813$, $p = 0.5613$.

(**F**) Mann-Whitney U test, $p = 0.6264$.

(**G**) Student's t test, $t_{(26)} = 0.0318$, $p = 0.9747$.

**Supplemental Figure 11**

(**A**) Student's *t* test, $t_{(16)} = 0.0035$, $p = 0.9972$.

(**B**) Student's *t* test, $t_{(14)} = 3.722$, $p = 0.0023$.

(**C**) Mann-Whitney U test, $p = 0.7374$.

(**D**) Student's *t* test, $t_{(24)} = 1.509$, $p = 0.1444$.

**Supplemental Figure 12**

(**B**) Student's *t* test, $t_{(16)} = 0.1964$, $p = 0.8467$.

(**C**) Student's *t* test, $t_{(16)} = 0.0649$, $p = 0.949$.

(**E**) Student's *t* test, $t_{(16)} = 0.3241$, $p = 0.75$.

(**F**) Student's *t* test, $t_{(16)} = 0.0094$, $p = 0.9926$.

(**H**) Student's *t* test, $t_{(16)} = 0.2012$, $p = 0.8431$.

(**I**) Student's *t* test, $t_{(16)} = 0. 514$, $p = 0. 6143$.

(**K**) Student's *t* test, $t_{(16)} = 0.5447$, $p = 0.5935$.

(**L**) Mann-Whitney U test, $p < 0.0001$.

(**N**) Student's *t* test, $t_{(16)} = 0.575$, $p = 0.5733$.

(**O**) Student's *t* test, $t_{(16)} = 2.231$, $p = 0.0404$.

(**Q**) Student's *t* test, $t_{(16)} = 2.681$, $p = 0.0164$.

(**R**) Student's *t* test, $t_{(16)} = 3.599$, $p = 0.0024$.

**Supplemental Figure 13**

(**B**) Student's *t* test, $t_{(5)} = 35.76$, $p < 0.0001$.

(**C**) Student's *t* test, $t_{(17)} = 0.2048$, $p = 0.8401$.

(**D**) Student's *t* test, $t_{(17)} = 2.279$, $p = 0.0359$.

(**E**) Student's *t* test, $t_{(17)} = 2.203$, $p = 0.0417$.

(**F**) Mann-Whitney U test, $p = 0.0002$.
